# Waste iron as a robust and ecological catalyst for decomposition industrial dyes under UV irradiation

**DOI:** 10.1007/s11356-023-27124-9

**Published:** 2023-05-02

**Authors:** Dominika Ścieżyńska, Dominika Bury, Michał Jakubczak, Jan Bogacki, Agnieszka Jastrzębska, Piotr Marcinowski

**Affiliations:** 1 Faculty of Building Services, Hydro and Environmental Engineering, Warsaw University of Technology, Nowowiejska 20, Warsaw, Poland; 2 Faculty of Materials Science and Engineering, Warsaw University of Technology, Wołoska 141, 02-507 Warsaw, Poland

**Keywords:** Amaranth dye, Acid red 27, Fenton process, Waste iron, Heterogeneous catalyst, Waste reuse

## Abstract

In an era of increasing environmental awareness, it is very important to work towards eliminating or at least reducing as many harmful industrial substances as possible. However, the implementation of green chemistry methods for wastewater treatment can be difficult especially due to complexity, the high cost of reagents, and the required long process time. This paper focuses on using waste iron (WI) to remove two kinds of amaranth dye commonly used in industry. To enhance the process, UV irradiation and hydrogen peroxide were used. The novelty of the research was the use of efficient and reusable WI as a heterogeneous catalyst in the process. WI material characteristics was done before and after the process using scanning electron microscopy (SEM), Fourier transform infrared spectroscopy (FTIR), and X-ray fluorescence (XRF). Zeta potential, size characterization, circularity, and direct band gap were also determined. As a result of treatment complete decolorization of both dyes was achieved, as well as 99% absorbance removal after 15-min process time. The total organic carbon (TOC) decrease after 60-min process time was in the range from 86.6 to 89.8%. Modified pseudo-second-order reaction reflects obtained results of treatment efficiency. Treatment results, confirmed by WI material characterization, indicate satisfactory stability of the catalyst and good oxidation capacity.

**Supplementary Information:**

The online version contains supplementary material available at 10.1007/s11356-023-27124-9.

## Introduction

Growing environmental awareness obliges industries to reduce their negative impact on the environment (Friedrich 2016; Hayat et al. 2020; Ranasinghe & Javasooriya 2021). Integrating sustainability into their strategy may be based on green technologies that are designed to reduce releasing toxic substances into the environment thereby saving water resources (Kunal et al. 2020). One of such substances are dyes.

Various technologies are based on dyes since they introduce colorful features into final products (Hassaan & El Nemr 2017). However, dye leakages are often within industrial conditions (Bhatia et al. 2017). This allows dyes to enter the ecosystem, further disrupting the natural environment. Even a small amount (< 1 mg L^−1^) of dye present in the water can be noticeable due to the appearance of a color. In the aquatic environment, dyes reduce the photosynthetic capacity of aquatic organisms due to limiting their access to light (Rafaqat et al. 2022). Natural decomposition of industrial azo dyes occurs in sediments and anaerobic conditions. Consequently, mutagenic aromatic amines are potentially released, being highly detrimental to aquatic organisms (Al-Tohamy et al. 2022; Markandeya et al. 2022). The presence of oil-based dyes may increase the levels of chemical oxygen demand (COD) and interfere with oxygen transfer at the air-water interface, thus reducing the purification capacity of the water reservoir. Once reaching the soil environment, dyes block soil pores, resulting in reduced productivity or bioavailable compounds. Plants are further disrupted due to soil hardening and reduced root penetration ability (Moni & Bharagava et al. 2018). While leaking into drinking water intakes, dyes affect water quality leading to the propagation of bacteria and viruses. Dyes also contribute to corrosion, perforation, and leakage of sewage pipes (Markandeya et al. 2022).

Notably, dye products can exist both as single components and mixtures. Single dyes are commonly used for laboratory practice and tests and therefore are called model dyes. Dye mixtures are composed of several compounds, customized to obtain desired color and seamless integration with the technological process.

Therefore, different treatment methods should be used to remove dyes and prevent them from getting into the environment. Usman et al. studied the aniline blue dye adsorption on activated pomegranate peel. They observed a 90.78% adsorption rate at optimal conditions: temperature 30 °C, pH  4.0, pomegranate peel activated carbon dosage 0.1 g, dye concentration 30 mg L^−1^, and time 50 min (Usman et al. 2022). Also, bioremediation approaches, such as the enzymatic action of partially purified orange (*Citrus reticulata*) peroxidase for the degradation of industrially important dye Drimarene orange KGL, studied by Hafiz et al, have been proposed to dye treatment as an addition to chemical and physical methods (Hafiz et al. 2014). Furthermore, Bibi et al. carried out the degradation process of Remazol brilliant blue, by environment-friendly fabrication, from seed extracts of citrus lemon, zinc oxide nanoparticle (ZnO NPs), under sunlight irradiation. After only 60 min of incubation, the degradation of Remazol brilliant blue was maximum at 85.91%. Moreover, ZnO NPs retained their activity after five washings (Bibi et al. 2020).

So far, considerable attention is given to advanced oxidation processes (AOPs) since they have already been proven to be the most efficient in decomposing dyes (Atalay & Ersoz 2021; Ismail & Sakai 2022; Peramune et al. 2022; Sajjad & Mousa Al-zobai 2020). The reason is that AOPs rely on the generation of highly reactive, non-selective radicals that are strong oxidizing species (Chen et al. 2021; Tkaczyk et al. 2020; Trojanowicz 2020; Ścieżyńska et al. 2022). Since they exhibit strong oxidative properties, they can degrade different organic pollutants (Tkaczyk et al. 2020). The specific variant of AOPs is the Fenton process, which has already proven its efficiency in decomposing both soluble and insoluble dyes (Bastaruk & Alver 2019; Fernandes et al. 2018; Heidari et al. 2019; Xu et al. 2020). It is based on the generation of hydroxyl radicals (Eq. 1) due to the reaction of hydrogen peroxide with iron ions in an acidic environment (Tkaczyk et al. 2020).

1 $$Fe^{2+} +H_2O_2\to Fe^3++ HO^\bullet + OH^-$$

The classical Fenton in which metal salts are used as a catalyst involves homogeneous reactions. The use of homogeneous Fenton has several disadvantages, including the problem of catalyst separation and regeneration. Therefore, such reactions often require the constant addition of the catalyst necessary to sustain the reaction. This leads to an increase of metals in the process, causing difficulties in the final treatment and the formation of a large amount of sludge. Replacing the classical catalyst with solid, iron-containing catalysts, and introducing heterogeneous reactions, seems to be a partial solution to those problems. Applying a heterogeneous catalyst in the process generates less sludge than the classic one due to the heterocatalyst’s lower solubility, ease of separation from the reaction mixture, and reusability. As a result, the need for the disposal of wasted catalyst is decreased. Moreover, the heterocatalyst can be modified to improve its selectivity.

In the dye degradation processes, an additional reaction catalyst, irradiation, is used. Examples of such assistance are gamma radiation (Muneer et al. 2019), electron beam irradiation (Deogaonkar et al. 2019), or ultraviolet (UV) irradiation (Al-Mamun et al. 2019, Ali et al. 2022). In this work, UV irradiation was chosen to enhance the treatment process. The use of UV radiation support is another way to increase the reactivity of iron ions. This modification’s principal concept is the photoreduction of the formed in the process Fe^3+^ ions to Fe^2+^ ions, which results in the formation of additional HO^•^ radicals. Therefore, the depletion of Fe^2+^ ions does not inhibit the process. This photocatalysis involves photon excitation in semiconductors and valence electron knockout. The induced electron, along with the resulting electron-hole pairs, are the initiators for a series of chemical reactions that produce superoxide ions (O_2_^−^) and hydroxyl radicals (Kiran et al. 2019). Moreover, UV irradiation is often considered the cheapest option among the irradiation methods. It is relatively easy to operate and does not require any radioactive sources or specialized equipment.

Over many years, scientists have carried out a plethora of degradation tests of various dyes using the Fenton process and its many variations. For instance, Nadeem et. al studied the degradation of synzol red using heterogeneous photo-Fenton catalysts such as ZnFe_2_O_4_ and graphene oxide/ZnFe_2_O_4_ composite (Nadeen et al. 2020). Zhang et al. used a continuous-flow electro-Fenton reactor and iron-carbon granules packed bed to degrade a model dye, tartrazine (Zhang et al., 2019a, b). Wang et al. conducted fast and efficient degradation of azo dyes including amaranth dye using photo-Fenton reaction and 3D/2D Fe_2_O_3_/g-C_3_N_4_ heterojunction catalysts composed of three-dimensional flower-like Fe_2_O_3_ and two-dimensional sheet-like graphite-phase carbon nitride g-C_3_N_4_ (Wang et al. 2021b). Grassi et al. further used calcined water treatment plant sludge as an alternative heterogeneous photo-Fenton catalyst for amaranth food dye removal from an aqueous solution (Grassi et al. 2020). Additionally, different catalyst in the degradation of amaranth dye was investigated, e.g., Zhang et al. investigated the degradation of amaranth using oxone catalyzed by MIL-101-NH_2_ under visible light irradiation and achieved excellent treatment results (Zhang et al., 2019a, b). Furthermore, Benaissa et al. used BiVO_3_/g-C_3_N_4_ S-scheme heterojunction nanocomposite photocatalyst for hydrogen production and amaranth dye removal and obtained about 90% efficiency with slightly less efficiency after the sixth cycle (Benaissa et al. 2021). Also, in Morajkar et al. publication, a comparison of various photochemical and biological methods available for treating amaranth dye is given (Morajkar et al. 2019).

Altogether, the abovementioned examples give some positive impressions on the successful application of the Fenton process to decompose dyes. However, there is a serious challenge in bringing these processes to industrial conditions. It comes from the fact that industrial wastewater includes many dyes that exhibit various properties that fairly disrupt the lab-scale processes more often initially designed to decompose model dyes. The same dye or dye mixture may also have several trade names, making it difficult to accurately recognize a specific composition. Associated variables in properties include the choice of compounds, solvents, and additives (Nikfar & Jaberidoost 2014).

This problem may be solved by designing modern materials like the aforementioned. However, the large costs of these catalysts make it another challenge for industrial implementation, causing it way too difficult. In this context, the application of cheap catalysts made from waste is an interesting option, since they are reusable, environmentally friendly, and could meet the requirements of sustainable development.

In this work, the challenge of designing a facile, cheap, sustainable, and industrially viable heterogeneous catalyst for dyes decomposition was taken. So far, attention was brought to the use of waste material in catalysis, e.g., iron slag (Nasuha et al. 2021) or iron-containing zeolites (Bencheqroun et al. 2022). A material garnering growing interest is waste iron. Waste iron can be obtained from various industrial processes, e.g., steel production, electronics, chemical or automotive industries, mining and mineral processing, and wastewater treatment. The limitations of catalytic activity are related to the source and characteristics of the material. Furthermore, the efficiency of the Fenton-like process as the classical Fenton process can be influenced by several factors such as pH, temperature, and the initial concentration of the dye. Nguyen et al. studied the reutilization of Fe-containing tailings ore enriched by Fe^3+^ chloride as a heterogeneous Fenton catalyst for the decolorization of organic dyes. The color removal efficiency of the MB and RhB system was achieved from 94.19 to 99.94% after 30 min of contact time and from 90.89 to 99.83% after 60 min reaction (Nguyen et al. 2021). Wang et al.’s heterogeneous Fenton process was employed to degrade Orange G using waste iron oxide (labeled as BT4) as the catalyst in a three-phase fluidized bed reactor. Total organic carbon (TOC) removal of 78.9% was achieved at a 50 mg L^−1^ initial OG concentration, 25 mg L^−1^ H_2_O_2_ concentration, pH 3, and 6 g L^−1^ BT4 addition (Wang et al. 2015). Furthermore, Mensah et al. evaluated the performance of three solid iron wastes as an activator of persulfate for the degradation of methylene blue. Their study revealed that iron waste can be efficiently employed for persulfate activation in the suspended and immobilized modes and degradades dyes from solution (Mensah et al. 2022). Wu et al. perform the evaluation of analytic ozonation of organic pollutants from actual bio-treated dyeing and finishing wastewater with iron shavings. Results indicated that the process was favorable for engineering applications for the removal of organic pollutants (Wu et al. 2016). These examples demonstrate the waste iron potential as a low-cost and eco-friendly alternative to traditional Fenton catalysts for wastewater treatment.

Therefore, this paper focuses on using a heterogeneous catalyst, a cast iron filling (waste iron, WI) with UV irradiation, to increase process effectiveness. To prove its efficiency, two industrially used dyes: Amaranth E123 and Acid Amaranth were chosen to carry out the process. A critical requirement for the treatment process is that it can be practically implemented in an industrial plant. This requirement is met by the catalyst used. This approach is important and novel in the industrial context since in most approaches, novel catalysts are too expensive, being non-applicable in industry. The parameters of the WI were characterized in detail both before and after the treatment process using scanning electron microscopy (SEM), Fourier transform infrared spectroscopy (FTIR), and X-ray fluorescence (XRF). Zeta potential, size characterization, circularity, and direct band gap were also determined.

## Materials and methods

### Amaranth dyes

Two kinds of commercial amaranth dyes were chosen for this research as representative of reactive azo dyes used in industry and were sourced from a cosmetic factory located in central Poland. The first one, Amaranth E123 (AM E123, 100% 1-(4-sulfo-1-naphthylazo)-2-naphthol-3,6-disulfonic acid trisodium salt, identified also as Acid Red 27, Food Colours Perczak Sp. J., Piotrków Trybunalski, Poland) with empirical formula C_20_H_11_N_2_Na_3_O_10_S_3_ is frequently used in industry as a food coloring agent, for specially processed foods, cakes, jellies, jams, ice cream or alcoholic beverages, and red soft drinks. AM E123 is a water-soluble organic compound with dark red to purple color (Chung et al., 1992; Nikfar & Jaberidoost 2014). The second one is Acid Amaranth (AM ACID, which is AM E123 with fillers and application enhancers of unknown composition, Boruta-Zachem Kolor S.A., Bydgoszcz, Poland), a popular industrial dye used for coloring natural and synthetic fibers, leather, paper, phenol-formaldehyde resins, and cosmetics like hair dyes, lipsticks blushes, foundations, and others. Stock solutions were prepared from powder stock before use. All solutions were prepared using ultrapure water.

### Characterization of waste iron

The waste iron (WI, Fig. S1) chosen for this research was cast iron fillings collected from the ordinary machining process. Digital images of WI grains were obtained with a DMS1000 microscope. The morphology and surface properties of WI were analyzed using scanning electron microscopy (SEM, Zeiss Ultra Plus, Zeiss, San Diego, CA, USA), at 2.0 kV accelerating voltage with different magnifications. WI was applied to the surface of the carbon tape. Finally, the sample was sputter-coated with gold using BAL-TEC SCD 005.

The surface area and porosity of the sample were determined with physical nitrogen sorption utilizing Quadrasorb-SI (Quantachrome Instruments, Odelzhausen, Germany), equipped with a FloVac degasser (Anton Paar GmbH, Graz, Austria). The specific surface area (*S*_*BET*_) was analyzed with the Brunauer–Emmett–Teller method (BET), while the total pore volume (*V*_*pore*_) and average pore size (*D*_*pore*_) with the Barret–Joyner–Halenda (BJH) method. The adsorption and desorption processes were carried out in a liquid nitrogen bath at −195 °C.

The presence of iron in the samples was checked by X-ray fluorescence (XRF, PI 100, Polon-Izot, Warsaw, Poland), equipped with a rhodium (Rh) anode. The analysis was recorded with a silicon drift detector (SSD) with 125–140 eV resolution and a multilayer monochromator. The elemental composition and elemental mapping of iron samples were evaluated with Hitachi S3500N scanning electron microscope (SEM, Hitachi, Japan) equipped with energy-dispersive spectroscopy (EDS) attachment. Before the studies, samples were put onto sticky copper tape and coated with a thin layer of gold using a BAL-TEC SCD 005 sputter coater. The studies were carried out with an accelerating voltage of 15 eV.

The optical properties of WI, especially absorbance, transmittance, and diffuse reflectance (DRS) were analyzed with double-beam scanning by UV-Visible Spectrophotometer (Evolution 220, ThermoFisher Scientific, Waltham, MA, USA) using an integrating sphere. The measurements were prepared at the wavelength range of 220–1100 nm, with an integration time of 0.3 s, a resolution of 1 nm, and a scanning speed of 200 nm min^−1^. Obtained results were used to calculate the indirect band gap with Tauc’s plot method (Eq. 1).

2 $${(\alpha hv)}^r=B\left( hv-{E}_g\right)$$

where *α*, *B*, and *E*_*g*_ were the absorption coefficient, the band tailing parameter, and the band gap energy, respectively and r=2.

The zeta potential of the WI nanoparticles solution was measured with the NANO ZS ZEN3500 analyzer (Malvern Instruments, Malvern, UK), operating at a 173° angle and 25 °C. The analysis was prepared in a measuring DTS1060 cell in five replications. The device was equipped with a back-scattered light detector.

The shape and size of WI grain were studied by a dynamic image analyzer (Sentinel Pro, Micromeritics Inst. Corp., Norcross, GA, USA) using the peristaltic pump and stroboscope camera. The chosen statistical data parameters were equivalent circular area diameter model, which focused on diameter (ECAD) and circularity.

Surface of the catalyst was checked by the Fourier transform infrared spectroscopy (FTIR, Nicolet iS5 FTIR Spectrometer, Thermo Scientific, Waltham, MA, USA). The device was equipped with attenuated total reflectance (ATR) and a diamond crystal. The analysis involved freeze-drying of the samples in order to avoid interference from water.

Additionally, the analysis of process adsorption was conducted using nitrogen (N_2_) physical sorption isotherms, performed by a Quadrasorb-SI device (Quantachrome Instruments, USA).

### Dye removal process

The experiments were carried out on aqueous dye solutions. The photo-Fenton process was used to remove the dyes.

Dye decomposition was carried out in a 1.5 L glass reactor, filled with a 1 L sample, to which appropriate amounts of WI and hydrogen peroxide (Stanlab, Lublin, Poland) were added. The process was carried out at a strictly controlled pH. Each sample was stirred at 300 rpm on a magnetic stirrer (Heidolph MR3000, Schwabach, Germany) to keep the catalyst particles in a dispersed form and prevent them from the aggregate. The UV light irradiation was performed with medium pressure Fe/Co 400W lamps type HPA 400/30 SDC, with 94W UVA power (Philips, Amsterdam, The Netherlands), placed vertically above the reactor (more details in supplementary material, Fig. S2). After selected process times (5, 10, 15, 30, 60) reaction was terminated by increasing the pH to 9.0 with 3 M NaOH (POCh, Gliwice, Poland). The samples were left overnight for the decomposition of unreacted H_2_O_2_ and iron hydroxide sedimentation. In order to determine the stability of the catalyst (iron leaching tests), the iron content was determined using the 1,10-phenanthroline method. The light absorbance was measured at wavelength 530 nm using a UV-VIS spectrophotometer (Hach DR 6000 Ames, IA, USA). Total organic carbon (TOC) was determined with a TOC-L analyzer (Shimadzu, Kyoto, Japan) with an OCT-L8-port sampler (Shimadzu, Kyoto, Japan).

The dye decomposition kinetics concerning the TOC value was determined. ANOVA with a 0.05 significance level was used to study the magnitude of variability in the average concentrations of TOC, and absorbance light.

Finally, the possibility of catalyst reuse without additional regeneration was checked. After the process, the WI was separated magnetically and a new treatment cycle with a fresh dye sample was carried out with the same initial parameters (pH, H_2_O_2_ dose, and process time).

### Analysis of the process’ mechanism

The activity of hydroxyl radicals was checked using a commercial colorimetric test, containing the O_2_-sensitive fluorescent reagent. Singlet oxygen levels were determined using Singlet Oxygen Sensor Green fluorescent reagent (Thermo Fisher Scientific, Waltham, MA, USA). The intensity of fluorescence was measured after 5 min of the photo-Fenton process under UV light irradiation. Thus, the 250 μL of dyes after the process were transferred into the multiwall plate. Next, the reagent was added to the sample to obtain a final concentration of 5.5 μM of colorimetric test solutions. Prepared samples were incubated in dark for 60 min, and the fluorescence intensity was measured using a microplate reader (Infinite 200 PRO, Tecan, Männedorf, Switzerland), on the excitation and emission of 485 and 520 nm, respectively.

### Waste iron diffusion test

The stability of WI was evaluated with the diffusion method. For this purpose, Gram-negative and Gram-positive bacteria and yeast selected from American Type Culture Collection (ATCC) were used. Among bacteria *Escherichia coli* (ATCC 10799), *Staphylococcus aureus* (ATCC 29213), and *Bacillus subtilis* (ATCC 11774) were used. Additionally, the yeast *Candida albicans* (ATCC 14053) was chosen as the representative fungi.

In the first step, a fresh, 24-h suspension (0.5 McFarland) of selected microorganisms in phosphate-buffered saline (PBS) was prepared. Next, nutrient solid agar medium (Merck, Kenilworth, NJ, USA) was inoculated by streaking with the sterile cotton swab containing the inoculum. After letting the medium dry for 10 min, the investigated materials (about 30 mg) were transferred to their surface with a sterile spatula. To ensure no overlapping of diffusion zones within the samples, samples were placed at least 24 mm from each other and 10 to 15 mm from the edge of the Petri plate. In the final step, prepared samples were incubated for 24 h at 37 °C. After incubation, photos of Petri dishes and measured diffusion zones were taken with ImageJ software (National Institutes of Health and the Laboratory for Optical and Computational Instrumentation, USA). The final result was calculated on the basis on ten measurement repetitions. Such an approach also allowed to calculate of the standard deviation (SD) and to estimate the measurement error.

## Results

### Waste iron characterization

The use of WI as an effective catalyst in the modified Fenton process for removing dyes is following the trend of using green chemistry in industrial development. In addition, the use of waste material is a long-awaited innovation and will lead to the resolution of real environmental problems. Working towards the treatment of wastewater containing dyes, their decreased release into the environment will consequently lead to the elimination of these pollutants. As a result, this will simultaneously eliminate at least some of the hazardous substances that have additional mutagenic and toxic properties, which dyes can be a source of. Therefore, the selection and characterization of a catalyst candidate for a modified Fenton process is a key element when it comes to final process efficiency.

**Fig. 1:**
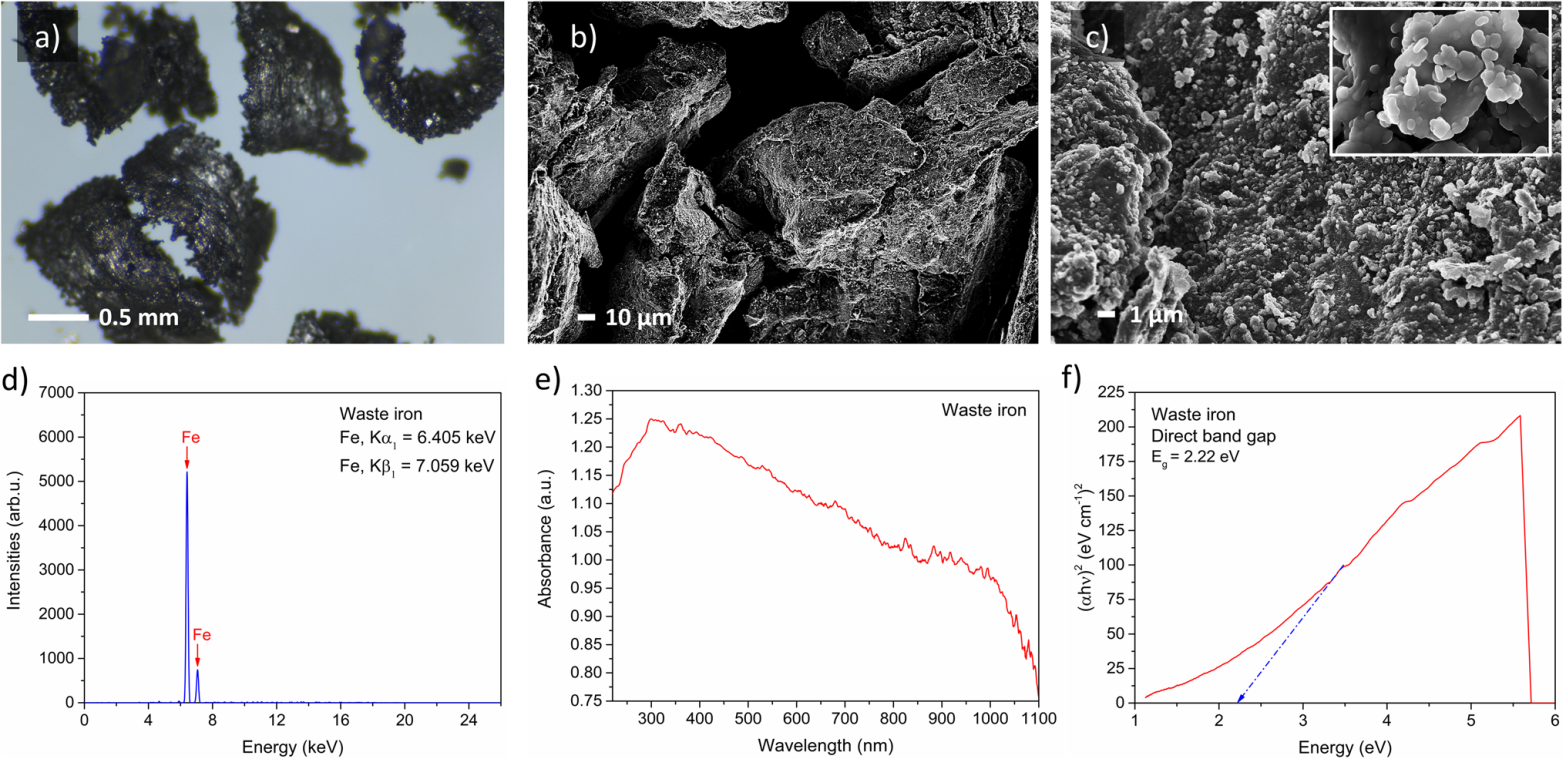
Waste iron (WI) characterization results comprising **a** digital images of swarf, **b**, **c** SEM images of swarf, **d** XRF pattern, **e** absorbance in 220–1100 nm wavelength, and **f** direct band gap derived from Tauc’s method

Firstly, the size, morphology, and structure of the materials were analyzed and are shown in Fig. 1. The WI grains were prepared in the form of the swarf approximately 1 mm wide and more than 1.5 mm in length (Fig. 1a). The results of a SEM analysis showed an irregular and smooth surface of grains without additional compounds (Fig. 1b). Interestingly, the minimal amount of the spherical nano-oxides and micro-oxides on the material surface were observed (Fig. 1c). The presence of nano-oxides suggests the slow oxidation process of iron.

Secondly, the composition of the samples was analyzed with XRF, as shown in Fig. 1d. Obtained results indicated the presence of iron on the grains without additional ingredients. Thus, the sample was characterized by high purity. As a result, WI could be perfect for utilization in the process.

**Fig. 2:**
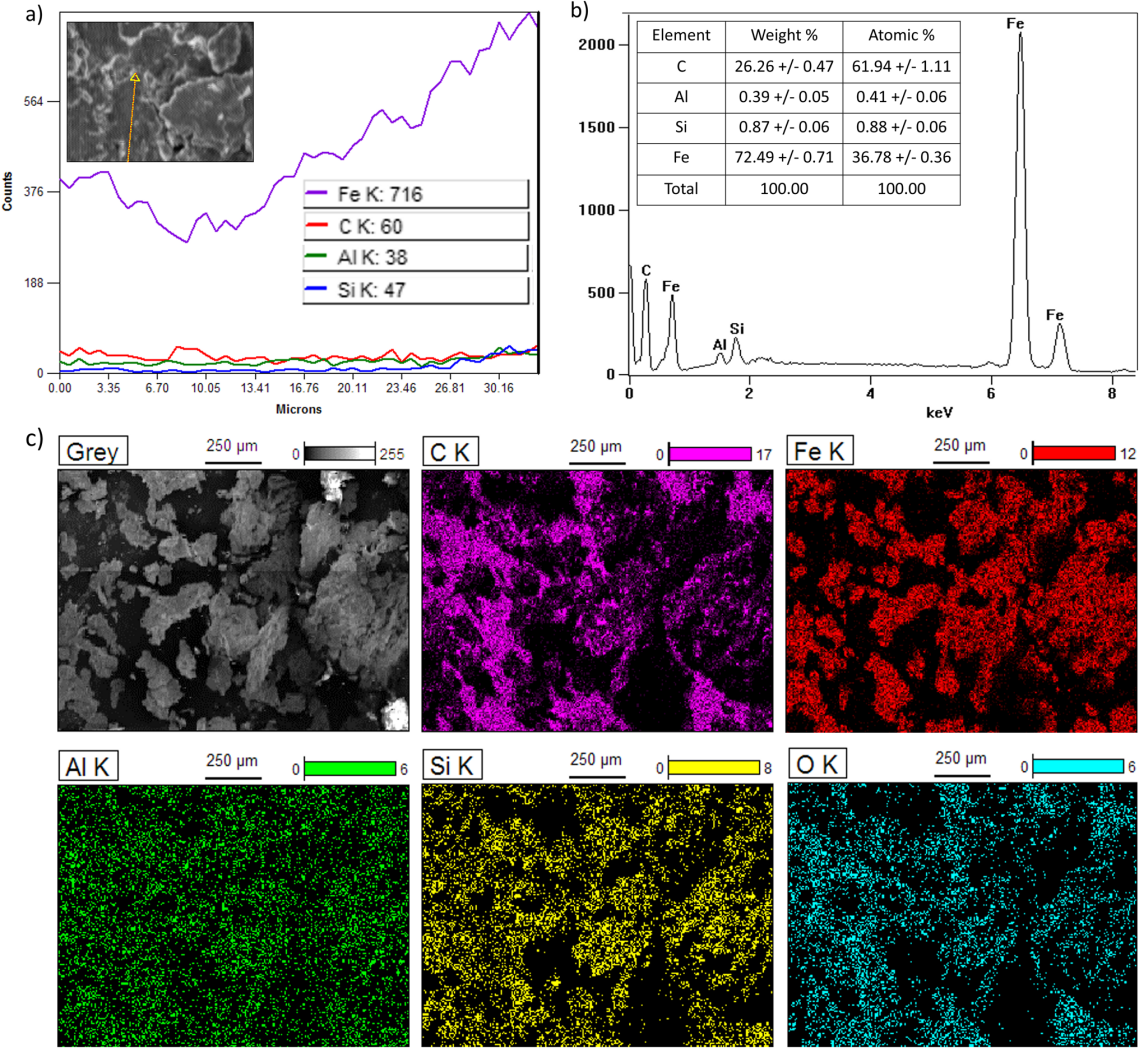
EDS analysis results: **a** at a point of a grain, **b** along the grain, **c** analysis of several grains with catalysts mapping

The EDS analysis results were showed in a few combinations - at a point of grain, along with the one and analysis of several grains with mapping of catalysts (Fig. 2 a, b, and c, respectively). The main catalyst component was iron, which constitutes more than 70% of the material. Additionally, a minimal amount of silicon was observed. Also, the components from sample preparation, such as carbon and aluminum were observed between the iron grains. In addition, the catalyst mapping showed the oxygen presence, located on the catalyst surface.

Next, specific surface area and porosity studies were carried out with the physical nitrogen sorption method. According our previous research, we noticed an excellent surface area equal to 42.44 m^2^ g^−1^, larger than detected in typical iron materials. Results were compared with Jozwiak et al. analysis of surface areas (Jozwiak et al. 2007). Scientists observed significantly smaller surface areas as 34.5, 21.9, 23.5, and 5.6 m^2^ g^−1^ for goethite, ferrihydrite, hematite, and magnetite, respectively. Moreover, an average material’s pore size of 11.0 nm and a mean pore radius of 2.0 nm were observed.

The following study also included an analysis of WI optical properties, especially absorbance (Fig. 1e) and direct bandgap (Fig. 1f). The WI had an optical absorption edge of approximately 300 nm, which is caused by the intrinsic absorption band affected by bandgap transition. The WI was characterized by total absorption in the visible light range (300–1000 nm). The extrapolated band gap value was 2.22 eV, similar to popular kinds of irons, especially hematite, magnetite, and goethite (2.1–2.2 eV) (Gardner et al. 2011; Millaty Abadiah et al. 2019). Thus, WI has a favorable band gap for UV harvesting.

Therefore, it was confirmed that WI has excellent properties such as unique structure, high purity, and amazing optical properties, thus it could potentially be used as the photo-Fenton process catalyst, under UV-light irradiation.

### Photo-Fenton process

Aqueous working solutions of both test dyes were prepared. The concentration of dyes 50 mg L^-1^ in the solution was selected based on data from the factory. The content of organic substances was checked using TOC. The initial TOC concentrations were 14.57 mg L^−1^ and 20.58 mg L^−1^ for AM ACID and AM E123, respectively. This indicates the inorganic nature of the fillers used in AM ACID. The initial light absorbance was 3.097 and 3.118 for AM ACID and AM E123, respectively. The prepared solutions were treated with the photo-Fenton process at pH 3, selected based on the literature review and the authors’ experience.

Processes with UV/H_2_O_2_, a catalyst with UV irradiation, UV irradiation alone for degradation for both AM ACID and AM E123 were conducted to verify the participation of independently occurring factors. The low efficiency of the processes using single components is due to the occurrence of minor processes during them, i.e., photolysis, adsorption, and oxidation (Table 1). The highest degradation results were achieved when all components were used together (1.95 mg L^−1^ for AM ACID and 2.098 mg L^−1^ for AM E123), and a modified Fenton process was carried out. Therefore, the use of all modifications together has a synergistic effect on efficiency and is associated with the greater generation of reactive radicals.

**Table 1:** Efficiency of processes using single components: UV/H_2_O_2_ with H_2_O_2_ dose 400 mg L^−1^, pH 3; time 60 min; catalyst with UV irradiation with WI dose of 1000 mg L^−1^ and 500 mg L^−1^ for AM ACID and AM E123, respectively, and pH 3; UV alone

	UV/H_2_O_2_		UV + catalyst		UV alone	
	530 nm absorbance	TOC [mg L^−1^]	530 nm absorbance	TOC [mg L^−1^]	530 nm absorbance	TOC [mg L^−1^]
AM ACID	0.052	10.21	3.063	10	3.058	10.03
AM E123	0.865	19.5	1.243	17.54	3.101	16.65

The first step of the research was the optimization of the treatment process parameters, which can improve the effectiveness and efficiency of dye removal and what is more, decrease reagent usage (Adachi et al. 2022; Blanco et al. 2014; Salgado et al. 2020). The optimal doses of reagents used in the research were selected based on the TOC results.

Preliminary dye degradation tests were carried out to determine the optimal catalyst dosage. Various doses of catalysts were checked after 15 min of irradiation under UV light, and an H_2_O_2_ dose of 400 mg L^−1^ and pH 3. Additionally, the dark Fenton efficiency was verified. For further study doses with the greatest reduction in TOC were selected. Eventually, after the UV irradiation-assisted process, a more significant decrease in TOC was obtained (2.5 mg L^−1^ for AM ACID and 5.61 mg L^−1^ for AM E123) than in the dark Fenton process (7.46 mg L^−1^ for AM ACID and 10.84 mg L^−1^ for AM E123). Therefore, dark Fenton was discontinued in further experiments and no kinetics calculations were performed for this process.

The selected WI doses were 1000 mg L^−1^ and 500 mg L^−1^ for AM ACID and AM E123, respectively (Table 2).

**Table 2:** TOC results after the process with different WI doses, with H_2_O_2_ dose 400 mg L^−1^, pH 3, time 15 min, and UV irradiation

	WI dose [mg]				
	200	500	1000	1500	Dark
AM ACID	6.22	4.47	2.5	4.84	7.46
AM E123	8.63	5.51	5.74	-	10.84

At the same time, the decolorization rate was 99%. Higher catalyst doses did not result in increased treatment effects, the bigger amount of dissolved Fe^2+^ ions acts as a radical scavenger (Eqs. 3, 4, 5, and 6) (Rayhani et al. 2019; Vorontsov 2019):

3 $$Fe^0\to Fe^{2+}+2e^-$$

4 $$Fe^{2+}+ HO^\bullet \to Fe^{3+}+ OH^-$$

5 $$Fe^{2+}+ HOO^\bullet \to Fe^{3+}+H_2O_2$$

6 $$Fe^{3+}+ HOO^\bullet \to Fe^{2+}+O_2+H^+$$

Moreover, it is possible that as catalyst doses are higher, the reaction mixture turbidity increases and limits the mixture’s light penetration. Light limitation overrides the increased amount of electron-pairs formation that would accelerate the reaction rate. As a result, catalyst doses that are higher than the determined optimum contribution to the reaction deceleration (Saeed et al. 2022).

**Fig. 3:**
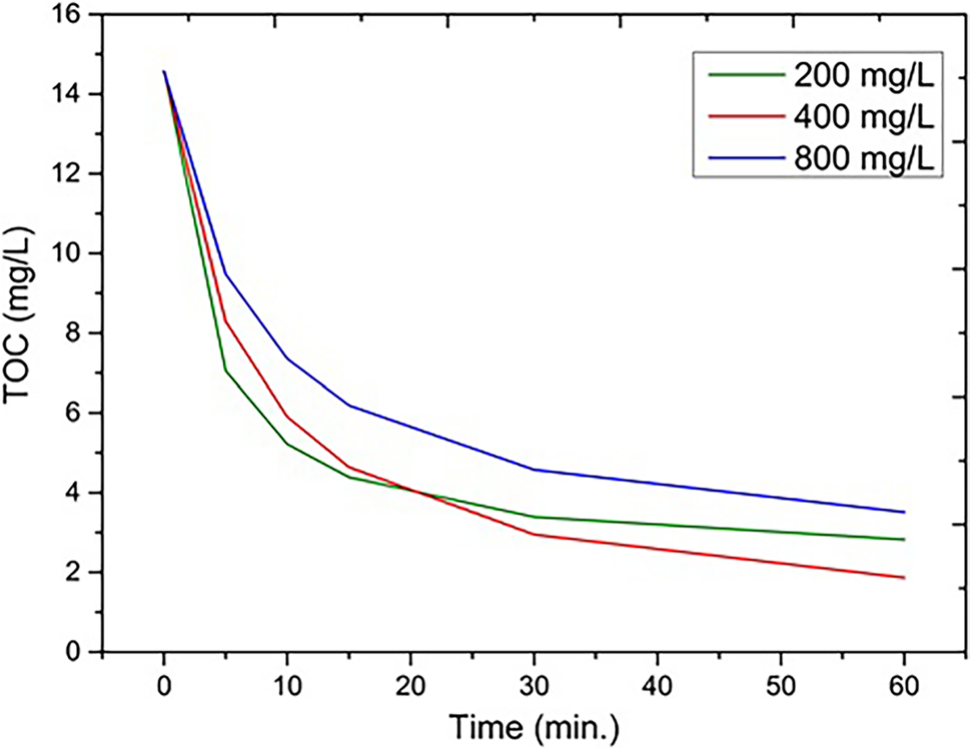
AM ACID decomposition through the UV/ZVI/H_2_O_2_ process, for selected H_2_O_2_ doses, and 1000 mg L^−1^ WI, pH 3.0

The selected H_2_O_2_ optimal dose was 400 mg L^−1^ with an efficiency of 89.8% and 86.6% in TOC removal for AM E123 and AM ACID, respectively. The use of other amounts of H_2_O_2_, 200 and 800 mg L^−1^, did not result in an increased treatment effect (Fig. 3).

The dose of 200 mg L^−1^ was too low, and the treatment efficiency was 85% TOC reduction for AM E123 and 80% for AM ACID. Rapid consumption and depletion of the substrate and the consequent slowing of the degradation process were observed. In contrast, the use of a dose of 800 mg L^−1^ resulted in an 86.6% for AM E123 and 76.7% for AM ACID in TOC decrease, which was associated with the introduction of excess hydrogen peroxide into the solution, which became an inhibitor of the reaction by the recombination of the excess hydroxyl radicals generated the unwanted hydroxyl radical reaction pathways [Eqs. 7, 8, 9, and 10]. Undesirable hydroxyl radicals’ pathways lead to their mutual scavenging or the formation of radicals with a much lower oxidative potential (Naeem et al. 2018; Rayhani et al. 2019; Vorontsov 2019):

7 $$HO^\bullet +H_2O_2\to HO_2^\bullet +H_2O$$

8 $$Fe^{2+} aq+ HO^\bullet \to Fe^{3+} OHaq,$$

9 $$HO^\bullet + HO^\bullet \to H_2O_2,$$

10 $$HOO^\bullet + HO^\bullet \to H_2O+O_2,$$

The excess H_2_O_2_ can lead to byproducts formation (such as iron oxides), which accumulates on the catalyst surface and can contribute to decreased process efficiency over time. Also, the aforementioned turbidity caused by the catalyst solubility during the process can be enhanced and promote deceleration and decreased efficiency of the process.

For an effective treatment process, a suitable reaction environment is required (Mansoorian et al. 2014; Maroudas et al. 2021; Teymari et al. 2020). After carrying out the process at different pH: 3, 4, 5, and 6, decreasing efficiency of dye degradation in solution was observed respectively. TOC reduction in the AM E123 treatment process decreased from over 89 to 11.5% as the pH increased from 3 to 6, respectively. In the case of AM ACID at pH 3, the TOC reduction was more than 86.6%, and at pH 6 it was 17.5%. Also, a strongly reduced decolorization effect with increasing pH was shown in absorbance results (Table 3).

**Table 3:** Absorbance results at 530 nm wavelength at different pH ranges and the process condition of H_2_O_2_ dose 400 mg L^−1^, pH 3, duration time 60 min, WI dose of 1000 mg L^−1^ and 500 mg L^−1^ for AM ACID and AM E123, respectively, combined with TOC [mg L^−1^] results

	pH 3		pH 4		pH 5		pH 6	
	Absorbance	TOC	Absorbance	TOC	Absorbance	TOC	Absorbance	TOC
AM ACID	0.022	1.95	0.021	3.33	1.308	10.81	1.430	12.02
AM E123	0.004	2.1	0.012	4.25	2.668	17.06	3.100	18.21

At acidic pH, the hydroxyl radical potential is 2.8 V, while under alkaline conditions the potential value decreases to 1.5 V. Moreover, the higher the pH value, the more affected the balance of photoactive iron compounds is. Under the process conditions, the two dominant photoactive forms are Fe^3+^ ions and Fe[OH]^2+^ species. At pH 3, they occur in almost equal proportions (Gomathi Devi et al. 2011). At both lowered and elevated pH, the balance of photoactive forms is disrupted, and thus, the efficiency of the process decreases. In addition, at higher pH, coagulation of Fe^3+^ ions begins and inhibits catalytic properties of Fe^2+^ ions, resulting in less radicals formed and dissociation of hydrogen peroxide to water and oxygen increase (Eq. 11) (Kremer 2021):

11 $$2\ H_2O_2\to O_2+2\ H_2O$$

Moreover, a drastic reduction in catalytic activity at pH above 4.0 occurs, due to the insolubility of Fe^3+^ species. It's because a corrosion layer develops on the ZVI surface during the process. The higher the solution pH, the greater the ZVI corrosion layer. The expanded corrosion layer slows the Fe^2+^ release causing a decrease in homogeneous radical formation. Due to the limitation of the homogeneous process, peroxide consumption mainly takes place in a heterogeneous process, on the catalyst surface (Donadelli et al. 2020).

**Fig. 4:**
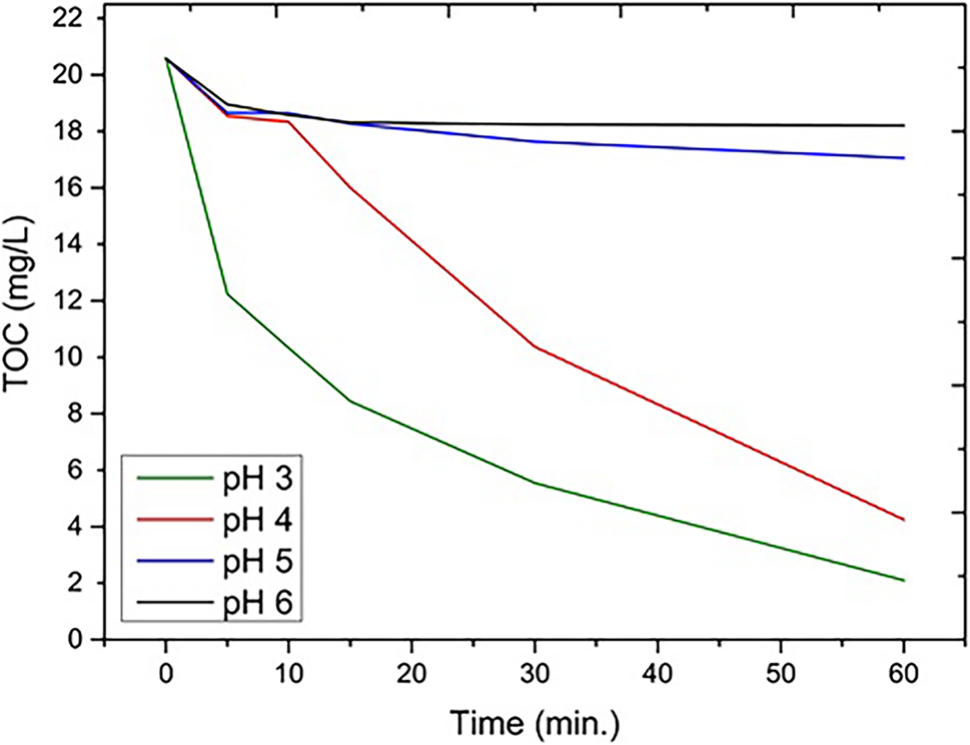
AM E123 decomposition through the UV/ZVI/H_2_O_2_ process, in different pH, and 500 mg L^−1^ WI

Therefore, pH 3 was chosen as the optimum reaction environment, in order to maintain the hydroxyl radical’s potential (Fig. 4).

**Fig. 5:**
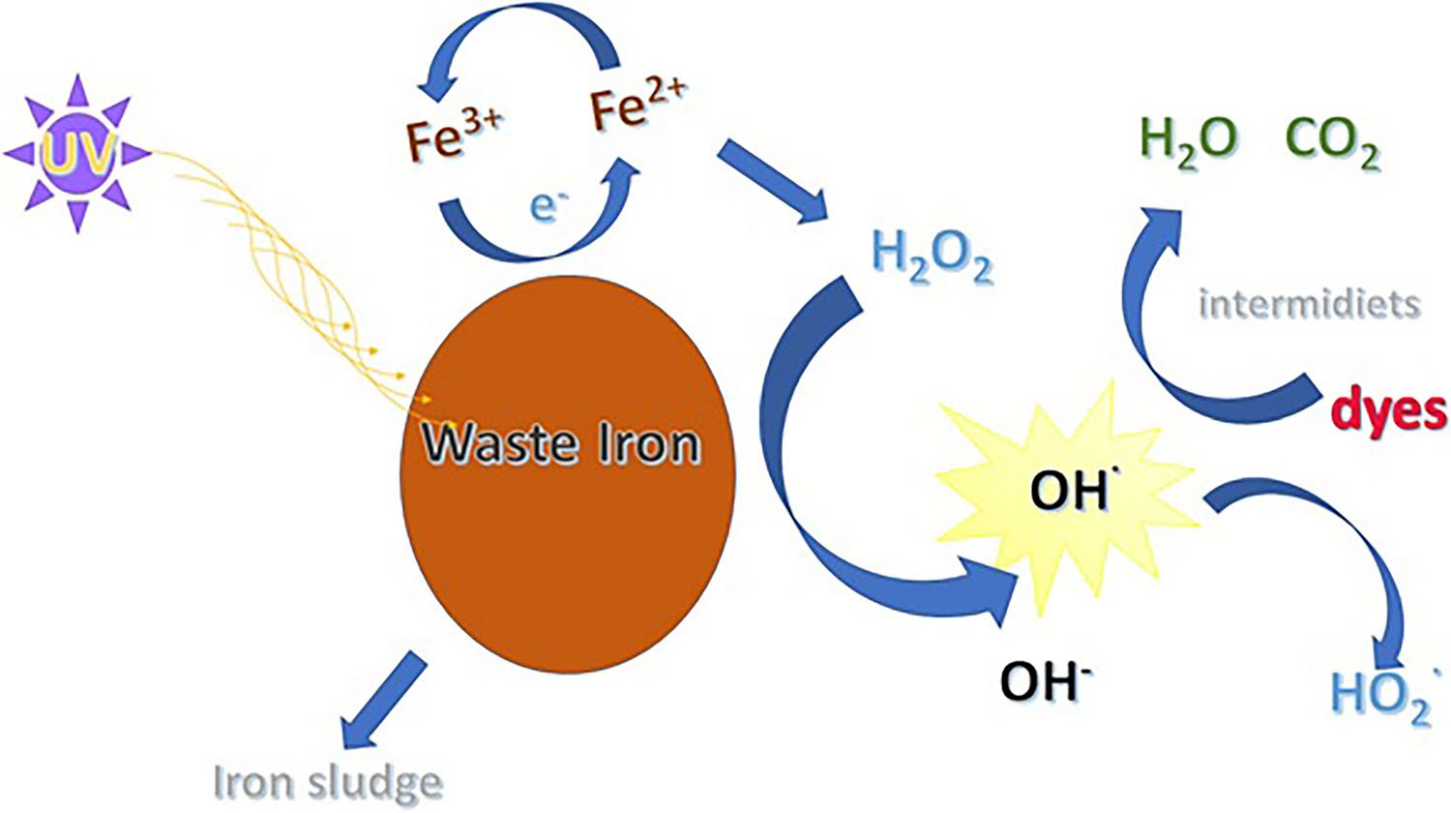
Catalytic process scheme

The catalytic process was very effective and the waste iron proved its efficiency in degrading a amaranth azo dyes. The catalytic scheme in Fig. 5 illustrates the process mechanism.

### Reaction kinetics and ANOVA

To describe the kinetics of cosmetic wastewater treatment processes for the purposes of the experiments, the following equations were used (Eqs. 12, 13, 14, and 15), (Marcinowski et al. 2020):

First-order reaction with respect to the value of TOC:

12 $$TOC= TOC_0\ast e^{- kt}$$

Second-order reaction with respect to the value of TOC:

13 $$TOC=\left( kt+1/ TOC_0\right)^{-1}$$

Modification of the first-order reaction with respect to the value of TOC:

14 $$TOC=\left( TOC_0-b\right)\ast e^{- kt}+b$$

Modification of the second-order reaction with respect to the value of TOC

15 $$TOC=\left( kt+\left( TOC_0-b\right)^{-1}\right)^{-1}+b$$

where *TOC*_*0*_ is the initial amount of TOC in dyes, *t* is the time of the process, *k* is the photo-decolorization reaction rate constant, and *b* is the process correcting parameter for non-oxidizable substances

Modified pseudo-second-order reaction best reflects obtained results of treatment efficiency. The optimal process time was 60 min. Further running the process did not increase its efficiency, expressed as TOC removal. The fastest process occurred during the first 15 min. After this time, the response slows down and a significant reduction in TOC content is no longer observed. A graph showing the relationship between actual results and kinetic calculations is shown in Fig. S3.

The ANOVA method was used to study the magnitude of variability in the average concentrations of TOC and absorbance light. TOC concentrations depended on the processing time, which was more important for degradation efficiency than the dose of H_2_O_2_. This is because of the need for H_2_O_2_ to react with available Fe^2+^ ions and the time for regeneration of these ions. However, the pH of the solution had a more significant effect on the efficiency of the process than its time duration. Anova calculation results are shown in Figs. S4 and S5.

### Process influence on catalyst surface properties

The influence of the photo-Fenton process in various pH values and for different reagent doses on the structure and properties of the WI under UV light irradiation were analyzed. Moreover, the catalyst’s properties after the process with both kinds of dyes were compared. The study of changes in the catalyst parameters allowed for a partial recognition of the process mechanism. The prepared research was extremely important and caused the reuse of the materials for the decomposition of dyes and using methods in the industry.

**Fig. 6:**
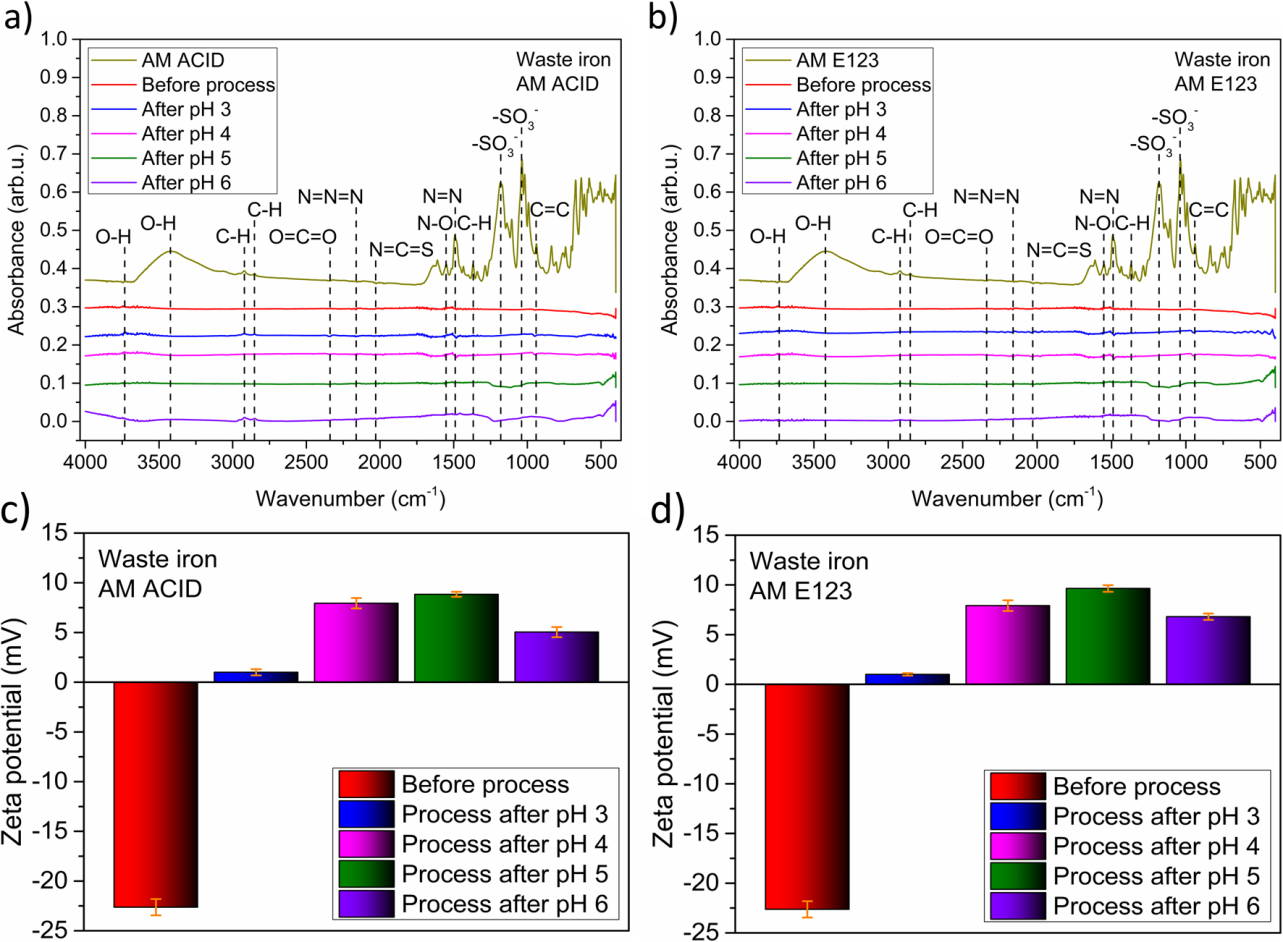
The catalysts surface analysis with **a** AM ACID and **b** AM E123 and the stability with **c** AM ACID and **d** AM E123 after 60 min of photo-Fenton process under UV light irradiation

Firstly, the surface of the catalyst with Fourier transform infrared spectroscopy (FTIR) was analyzed and showed for catalyst after the process with AM ACID and AM E123 (Fig. 6 a and b, respectively). To analyze the adsorption of dyes on the WI surface, rocking vibrations for dyes and catalysts before process and after the process in various pH values were recorded. Interestingly, the same results for materials before and after the process in both kinds of dyes were detected. What’s more, the energy vibrations characteristics for dyes were not noticed for samples after the process. These results suggest that dyes and their decomposition products did not permanently adsorb on the catalyst’s surface. Both dyes behave in the same way under the tested conditions. Moreover, the adsorption process is associated with the material active surface. This was elucidated already in previous work, strictly concentrated on waste iron material characterization in Novel photo-Fenton nanocomposite catalyst based on waste iron-Ti_3_C_2_T_*x*_ MXene for efficient water decontamination (2023, in press). Results show 0.6324 m^2^ g^−1^ of active surface area and the total surface of the pores volume of 0.308 m^2^ g^−1^. Additionally, the adsorption process with waste iron combined with nanostructures as catalysts were prepared. The adsorption process only supported the Fenton process and did not affect a significant impact on the excellent results of dye decomposition. Simultaneously, Lv et al. showed minimal MB decomposition during the reaction in 285 min, at initial pH 3 with 40 mM H_2_O_2_, 1 g L^−1^ Fe_2_O_3_ dosage (Lv et al. 2015).

In the next step, Zeta potential analysis was studied at different pH values. The results after the process with AM ACID and AM E123 are shown in Fig. 6 c and d, respectively. The process with both dyes affected a similar decrease in catalyst stability. The smaller the negative value of the Zeta potential, the greater the stability of the material. The catalyst before the process was characterized by a Zeta potential value of − 23 mV, while after the process positive values of the parameter were recorded. Interestingly, low stability was detected for the catalysts after the photo-Fenton process in pH 3 (− 1 mV). What is more, slightly higher stability was detected with increasing pH value. We detected Zeta potential values 8, 9, and 5 mV for pH 4, 5, and 6, respectively, for materials after treatment AM ACID. Interestingly, the Zeta potential values 9, 10, and 6 mV for pH 4, 5, and 6, respectively, for samples after the process with AM E123. The catalyst stability changes could be the results of oxidation of the material’s surface and activity with hydrogen peroxide, during the photo-Fenton process.

Furthermore, the behavior of the dyes at different pH was verified. The observed absorption band decay at 530 nm in the UV-VIS spectrum is related to diazo bond breaking in subsequent Amaranth molecules and the destruction of the conjugated bond system (Table 3).

**Fig. 7:**
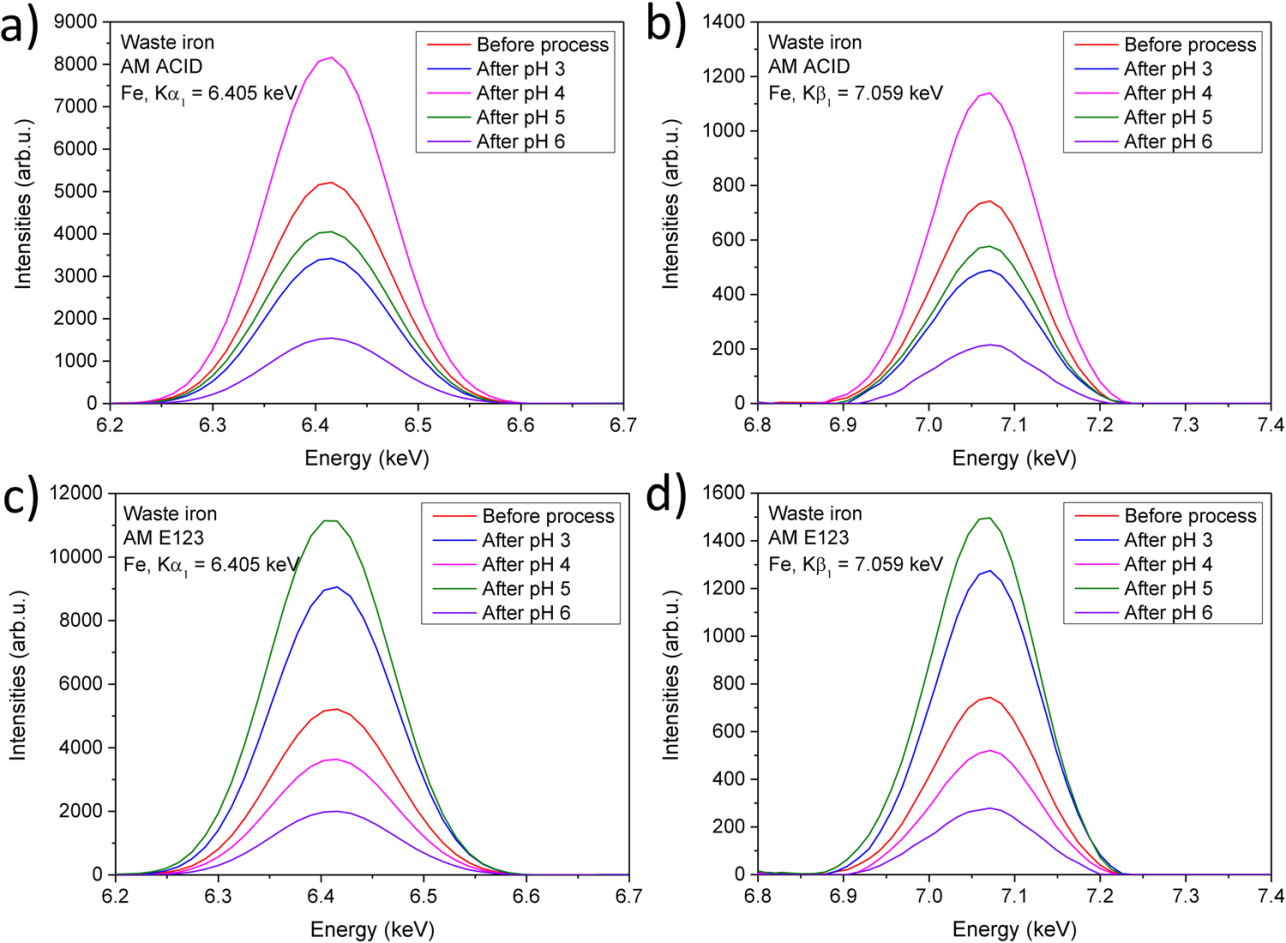
XRF analysis of the presence of iron in Kα_1_ = 6.405 keV after process with **a** AM ACID and **c** AM E123. XRF analysis of the presence of iron in Kβ_1_ = 7.068 keV after process with **b** AM ACID and **d** AM E123

In the subsequent step, the components of the WI after the photo-Fenton process were analyzed and compared to the results with materials before dyes treatment with XRF (Fig. 7). The presence of iron in all samples after the process was detected. These results suggest the possibility of utilizing WI grains following the photo-Fenton process. Therefore, the catalyst could be saved, resulting in lower operating costs and enhancing the possibility of industrial use.

**Fig. 8:**
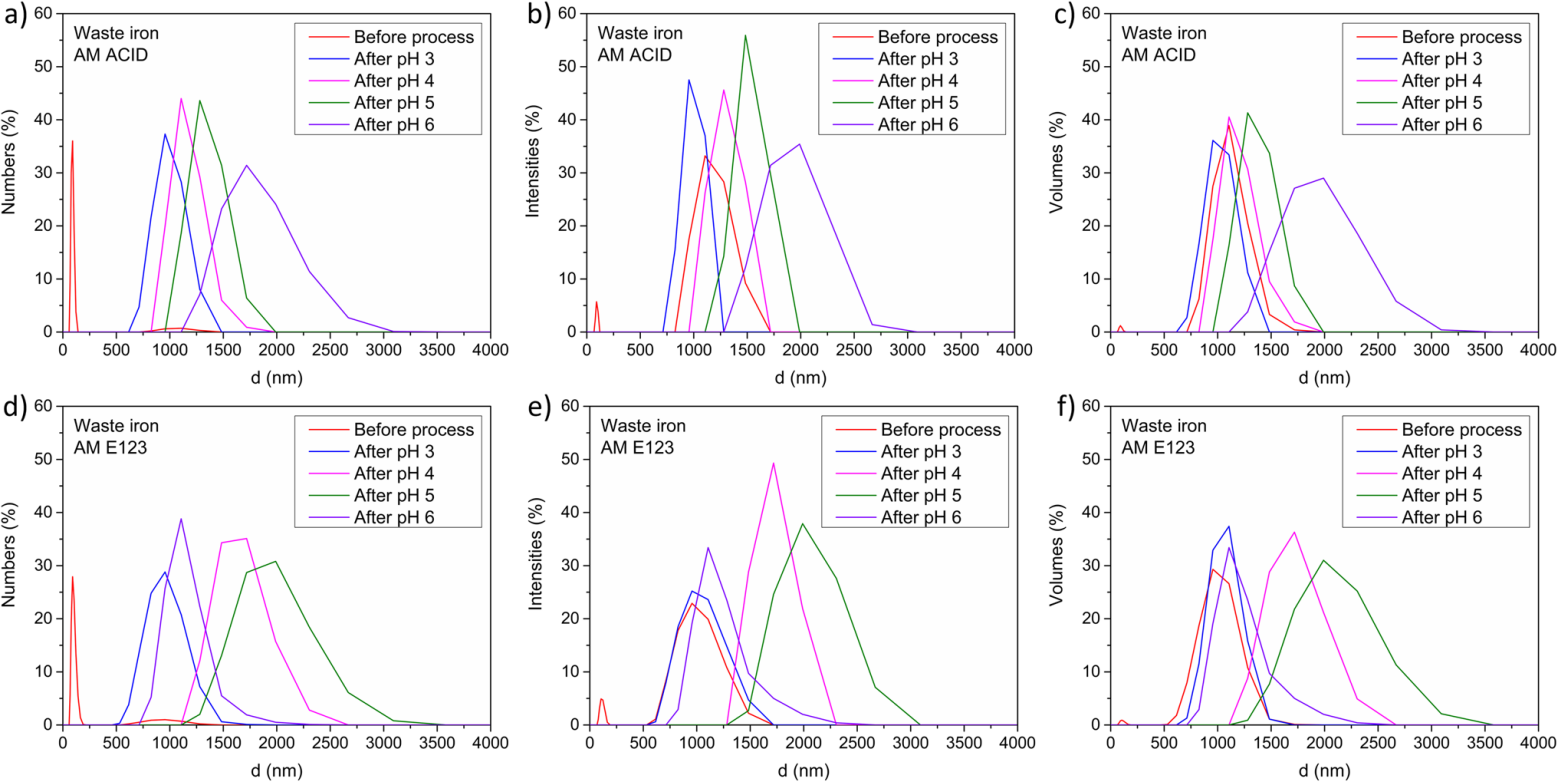
Size characterization results catalysts after process with AM ACID (**a**–**c**) and AM E123 (**d**–**f**)

**Fig. 9:**
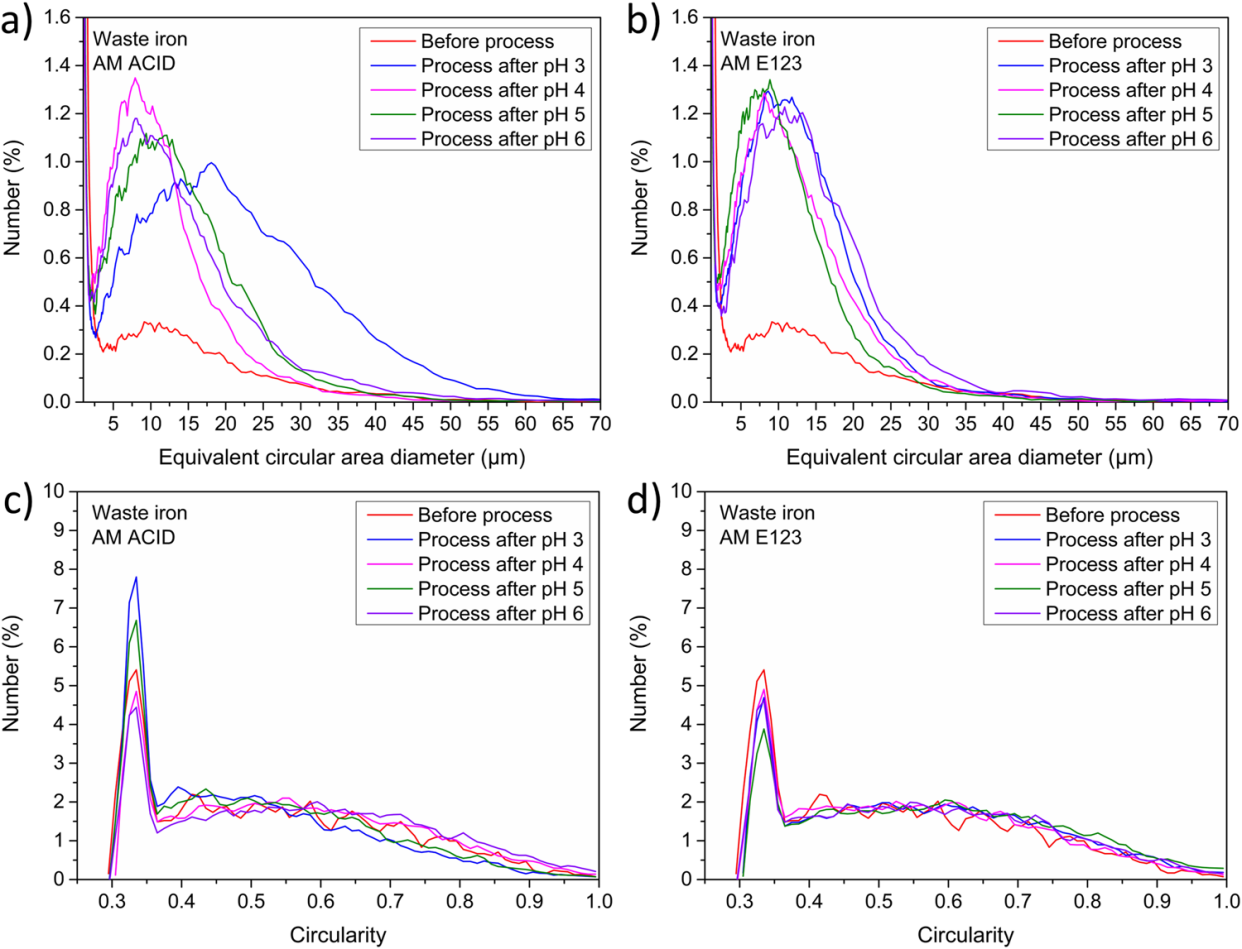
Comparison of shape parameters such as minimum equivalent circular area diameter for samples after process with **a** AM ACID and **b** AM E123. The circularity of material’s grain after methods with **c** AM ACID and **d** AM E123

The next part of the work included an analysis of the catalyst’s key morphological parameters such as the size and shape of materials after the process. The results are shown in Figs. 8 and 9. The highest hydrodynamic diameter of the grains was detected for samples AM E123 in pH 4 and 5 (from 1000 to 3500 nm) and slightly less in pH 3 and 6 (from 500 to 2000 nm) after treatment. Additionally, the higher intensity of equivalent circular area diameter for samples before the process for both kinds of amaranth (in the range from 5 to 50 μm) was detected. Thus, obtained results suggest that the process caused more agglomeration of catalysts. Moreover, the results of the circularity of all samples were identical and were in the range from 0.3 to 0.4.

**Fig. 10:**
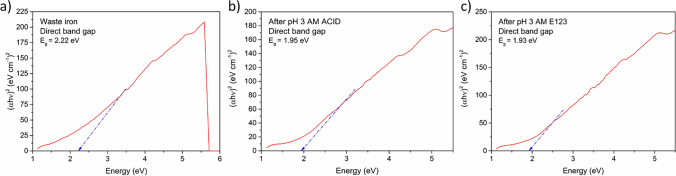
The direct band gap **a** before process and after 60 min of photo-Fenton process under UV light irradiation for **b** AM ACID and **c** AM E123

Finally, the changes in the optical properties of catalysts after the process with both kinds of dyes were analyzed. Thus, the absorbance results are shown in Fig. S6. The absorbance light did not change significantly before and after the process. Moreover, catalysts still could absorb light in all spectrums of visible light (from 300 to 1100 nm.). Obtained results were utilized to evaluate direct band gaps, which were imagined in Fig. 10. Interestingly, direct band gaps were slightly smaller after the process than before decomposition. The parameters checked on the pure catalyst were 2.2 eV for both dyes, while 1.93 and 1.95 eV for AM ACID and AM E123 degradation respectively were detected after the process. These results suggest that the catalyst has maintained its light absorption properties and can be reused in the following photo process.

### Waste iron diffusion test

**Fig. 11:**
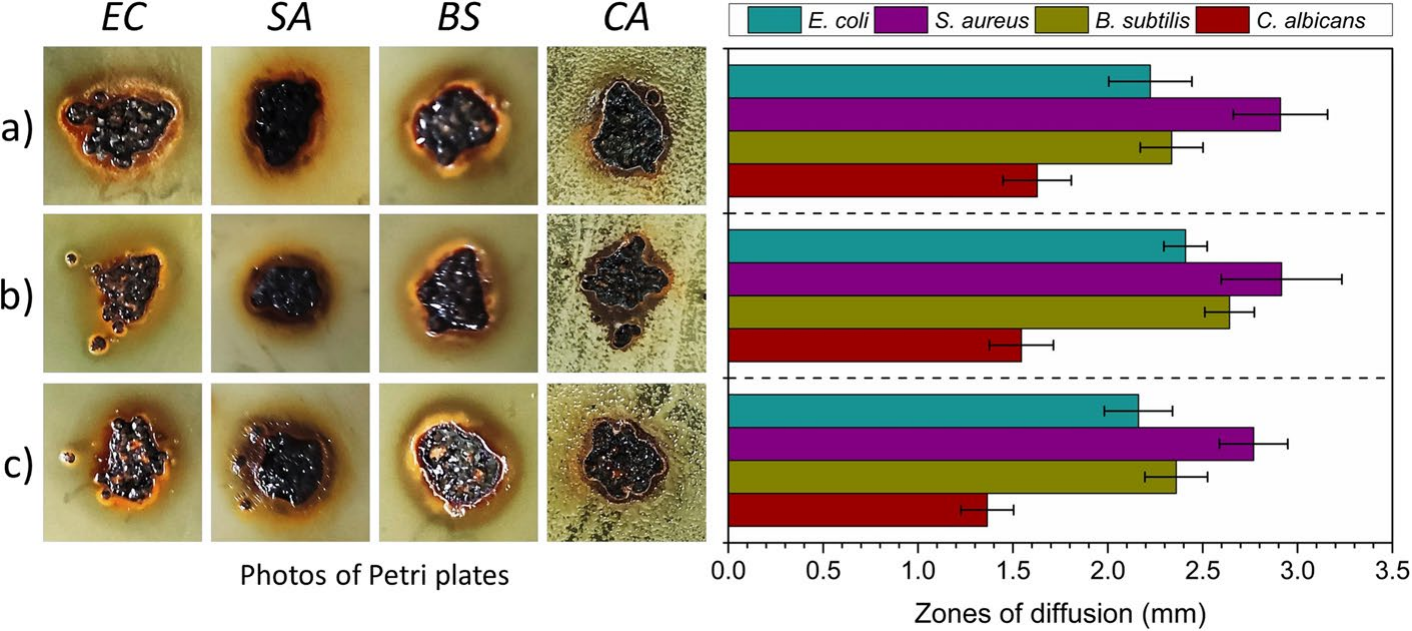
The results of diffusion test performed for **a** reference WI, **b** WI after treatment of AM ACID in pH 3, and **c** WI after treatment of AM E123 in pH 3. The test was performed with *E. coli* (EC), *S. aureus* (SA), *B. subtilis* (BS) bacteria, and yeast *C. albicans* (CA). The results were presented as photos of Petri plates showing zones of diffusion (left part of the figure), and measured zones of diffusion (right)

In the next part of the study, the stability of the catalyst was evaluated using a diffusion test and selected bacteria and yeast. The diffusion test works by allowing active substances, such as antibiotics or metal ions, to diffuse into an agar medium. Such substances prevent the growth of microorganisms on the surface of the growth medium, which causes a color change around the tested samples. Figure 11 shows the results of this diffusion test.

Based on the results, it was observed that the tested materials exhibited high stability. The observed diffusion zones of ginger were directly related to the oxidation of iron during incubation and the subsequent diffusion of iron ions into the agar medium. The largest diffusion zones were observed in the case of *S. aureus* bacteria, while the smallest were observed for the yeast *C. albicans*. However, the diffusion zones were very similar between the strains of microorganisms tested, indicating a low impact of the process on the catalyst and similar stability of the material before and after treatment. Several research groups have already confirmed the antibacterial properties of iron oxides based on their studies (Janani et al. 2021, Saqib et al. 2019, Irshad et al. 2017, Vasantharaj et al. 2019). It is known that different types of iron oxides are generated under various conditions and environments. For instance, magnetite (Fe_3_O_4_) can be formed in reducing conditions, while hematite (Fe_2_O_3_) is produced in oxidizing conditions. Additionally, goethite (FeO(OH)) and siderite (FeCO_3_) can be generated under specific environmental conditions (Cornell and Schwertmann 2003; Schwertmann and Cornell 1991). Interestingly, studies suggest that the antibacterial activity of Fe_3_O_4_ and iron nanoparticles may be more pronounced against Gram-negative bacteria because their cell walls are more porous, which allows for easier penetration of the nanoparticles (Vasantharaj et al. 2019). In contrast, the thicker and more complex cell walls of Gram-positive bacteria may provide better protection against the antibacterial effects of the particles. Thus, the differences in cell wall structure between Gram-positive and Gram-negative bacteria may contribute to the varying effectiveness of Fe_3_O_4_ and iron nanoparticles as antibacterial agents (Chapot-Chartier et al. 2014, Rucker et al. 2008). The difference in antibacterial activity between Gram-positive and Gram-negative bacteria may impact the effectiveness of the material as antibacterial agents. However, the stability of the material is unlikely to be significantly affected by this difference. This is because the same iron oxide and nanoparticles that act as an antibacterial agent are being generated, regardless of the specific bacteria being targeted.

Overall, the results of the diffusion test demonstrate the high stability of the catalyst and its ability to withstand the treatment process without significant degradation or loss of activity. This is an important finding, as it indicates that the catalyst can be used repeatedly over time without significant deterioration in its performance (Fig. 11).

### Reusability

**Fig. 12:**
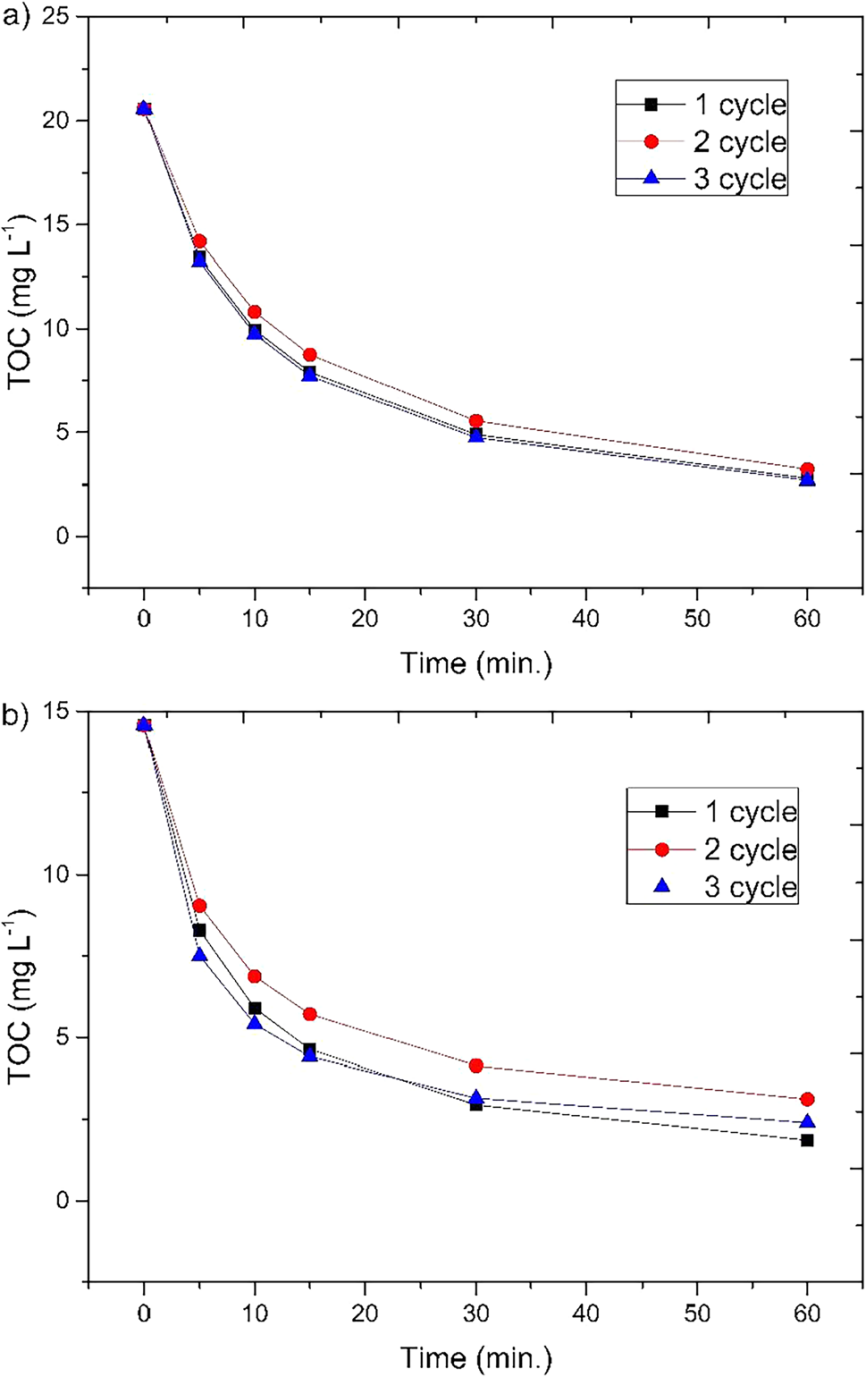
Reusability for 3 cycles with optimal parameters of 400 mg L^−1^ H_2_O_2_, pH 3, and UV irradiation: **a** with 500 mg WI and 50 mg L^−1^ AM E123, **b** with 1000 mg WI and 50 mg L^−1^ AM ACID

An important element of the present study was to check the possibility of reusing the material. The reusability of the catalyst is a parameter used to measure its stability and activity. It is also a key issue for practical application because of the operation costs (Wang et al. 2021a). The optimal condition described above was chosen to carry out the reuse processes. After each run, the WI was separated from the treated solution and transferred to the next one, with the same optimal initial values as in the previous run, without additional treatments. This procedure was repeated for three cycles, results are shown in Fig. 12. The process efficiency oscillated between 82.9–89.8 and 83.9–86.6% for AM E123 and AM ACID, respectively. The results indicate satisfactory stability of the catalyst, good oxidation capacity of the molecules, and a slight decline in both dyes’ degradation. Moreover, the decolorization in all processes was maintained at more than 99%.

Based on the dye degradation results after three cycles of material reuse, it can be concluded that waste iron has the potential to be used in the treatment process to a wider extent than presented in this paper. However, it should be noted that during the process iron leaching from the heterocatalyst occurs. The dissolved iron ions contribute to the catalytic activity, and therefore, some degree of iron leaching is necessary for the Fenton-like processes to work effectively. Nevertheless, this leaching can occur more readily at lower pH values. Excessive leaching can lead to environmental concerns and may also impact the efficiency and stability of the process. Therefore, it is important to monitor the dissolved iron present in the solution. For this purpose, the amount of remaining dissolved iron in the sample after treatment and sludge separation was measured. An increase in iron solubility was observed with the subsequent treatment cycle (from 36.32 to 38.32 mg L^−1^). This indicates the necessity of implementing a final coagulation process before wastewater discharge to meet legal requirements for sewage disposal.

### Possibility of practical application

The obtained treatment results are very promising; therefore, the potential possibility of implementing the research results in a practically existing installation can be considered. Numerous issues need to be resolved before potential deployment. The first is to conduct research, still on a laboratory scale, but using real industrial wastewater. Due to the presence of additional pollutant, a reduction in the efficiency of dye removal is expected due to competition with other organic compounds. In order to maintain a high degree of pollutants removal, the doses of reagents and the time of the process should probably be increased. Another problem is the transition from a laboratory scale to a semi-industrial scale to an industrial scale. At this stage, it is necessary to solve the issue of the optimal way of conducting the process, including the design of the installation and the development of the optimal way of using the catalyst. Here, the most prospective possibilities are probably the immobilization of the catalyst on the carrier, or the use of an effective method of separating the catalyst from wastewater, its regeneration, and reuse. In parallel, regardless of technological research, economic factors should be considered. Currently, the catalyst is prepared in small laboratory quantities, which is associated with significant cost-intensive production. On an industrial scale, the need for a catalyst would be much greater. With increased scale, the unit price of the catalyst would be much lower, but it may still be too high and constitute an economic barrier to the implementation of the new technology.

## Conclusion

In this work, the characterization of WI as a solid catalyst used for the degradation of AM ACID and AM E123 dyes was reported. WI catalyst reached high efficiency in the dye degradation after 60 min of treatment at pH 3, under UV irradiation coupled with hydrogen peroxide dosing 400 mg L^−1^. As a result of treatment complete decolorization of both dyes was achieved, as well as 99% absorbance removal after 15-min process time. The TOC decrease after 60-min process time was in the range from up to 89.8%. Furthermore, single component processes were significantly less effective due to the occurrence of minor treatment processes during them. The modified pseudo-second-order reaction reflects obtained results of treatment efficiency. ANOVA analysis indicated that TOC concentrations depended more on the processing time than H_2_O_2_ dose. However, the solution pH was more significant than the duration time.

The parameters of the used WI were characterized in detail both before and after treatment process, including SEM, surface area, porosity, BET, BJH, XRF, zeta potential, DRS, UV-VIS, FTIR, and ATR analysis. The adsorption process on the material surface was minimal and did not noticeably affect dye degradation. Moreover, observed absorption band decay at 530 nm in the UV-VIS spectrum confirmed the degradation of azo bonds.

The catalyst stability changed depending on the environmental pH, which was the result of oxidation of the material’s surface. Although, the diffusion test results demonstrate the high stability of the catalyst and its ability to withstand the treatment process without significant degradation or loss of activity. Moreover, the catalyst three reuse experiment shows the recyclable and stable performance of the process.

This all makes the WI a very promising catalyst, which can provide an efficient and cost-effective process for the degradation of organic contaminants in wastewater treatment processes. Further process implementation on an industrial scale can be considered after its limitations are resolved.

Based on the conducted research and determination of the conditions of applicability of WI to remove dyes from the water phase, it is planned to continue research on the actual wastewater from the factory, containing in addition to dyes other difficult to decompose and potentially harmful chemical compounds.

## Supplementary information

ESM 1: Figures S1–S6 (DOCX 2921 kb)
