# Supplementary material for: Waste iron as a robust and ecological catalyst for decomposition industrial dyes under UV irradiation
Source: Environ Sci Pollut Res Int. 2023 May 2;30(26):69024–41. doi: 10.1007/s11356-023-27124-9 (PMC10212823; doi:10.1007/s11356-023-27124-9)
Supplement: Supplementary file 1 — Figures S1–S6 (DOCX 2921 kb) [file 11356_2023_27124_MOESM1_ESM.docx]

***Supplementary Material***

**Waste iron as a robust and ecological catalyst for decomposition industrial dyes under UV irradiation**

***Dominika Ścieżyńska^1^, Dominika Bury^2^, Michał Jakubczak^2^, Jan Bogacki^1^*******, Agnieszka Jastrzębska^2^ and Piotr Marcinowski^1^***

*^1^ Warsaw University of Technology, Faculty of Building Services, Hydro and Environmental Engineering, Nowowiejska 20, Warsaw, Poland*

*^2^ Warsaw University of Technology, Faculty of Materials Science and Engineering, Wołoska 141, 02-507 Warsaw, Poland*

* jan.bogacki@pw.edu.pl@pw.edu.pl


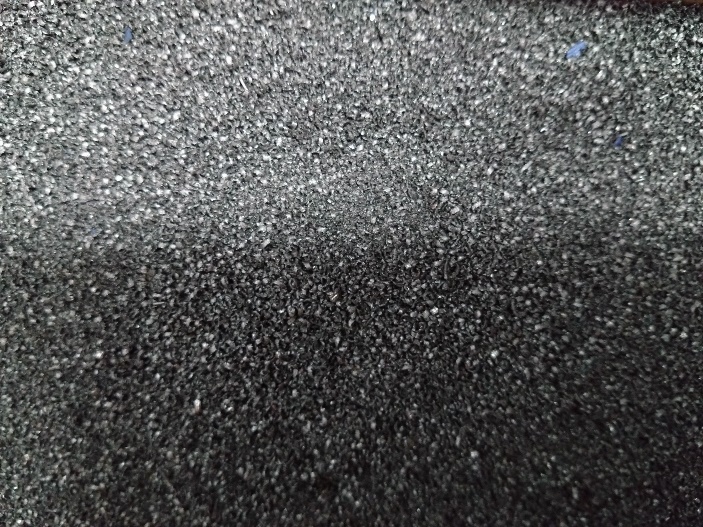

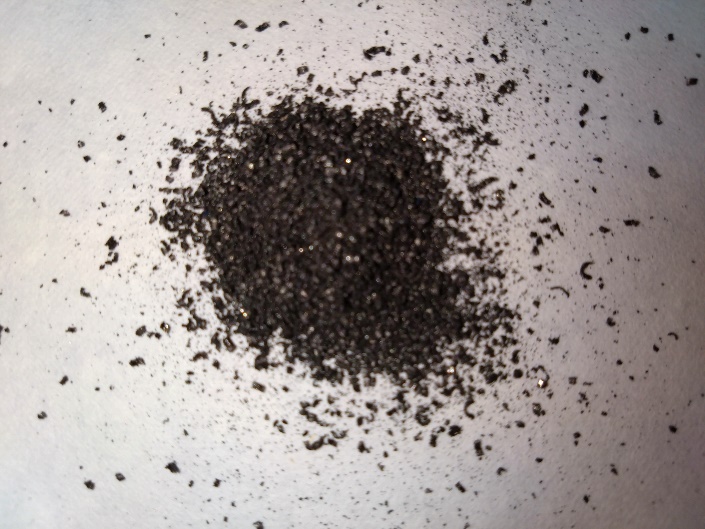


**Figure S1.** Waste iron from machining process.


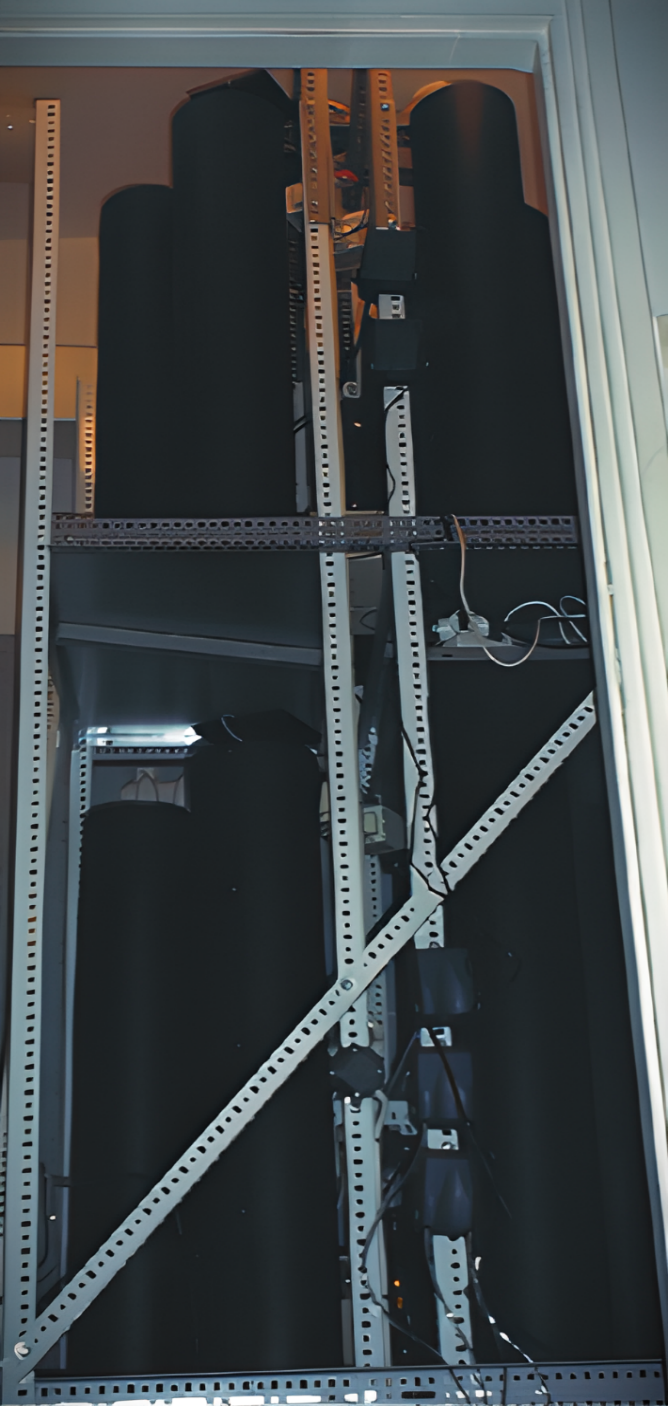

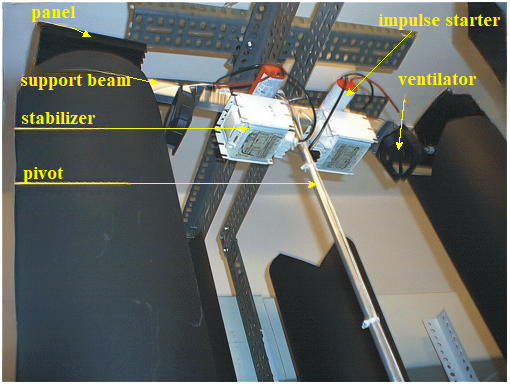

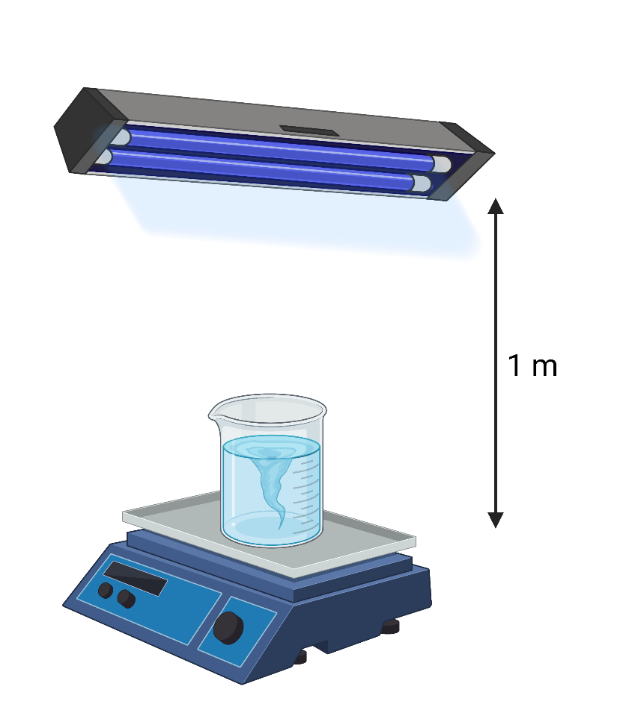


c)

b)

a)


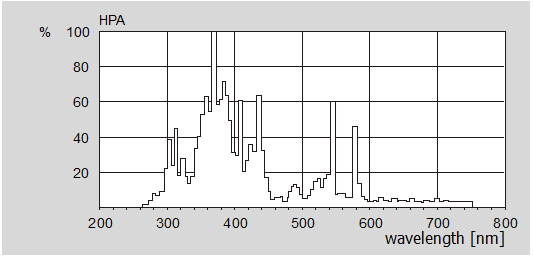


d)

**Figure S2: a)** UVA lamp placed vertically above reactor, **b)** visualization of the set elements**, c)** visualization of the reactors**, d)** the direct radiation spectrum of the HPA-type lamps

The irradiation test stand was composed of two levels (Figure S2c). The set-up main component were four medium-pressure lamps (iron and cobalt doped halogen lamps, PHILIPS, HPA 400/30 SDC). The direct radiation spectrum of the HPA-type lamps is shown in Figure S2d. The UV radiator was suspended under a R3 type panel. The panel reduced the influence of scattered and reflected radiation on the sample, which at the same time guaranteed the use of only direct radiation for the initiation of chemical transformations in the reactors. In addition, the panel together with the ventilator constituted the lamp cooling system. The panel, ventilator, slat terminals, lamp, and an SUZ-M 214 electronic impulse starter with BF/153 MagneTek stabilizer, were secured to an aluminum support beam. The two support beams were mounted in a single plane, perpendicular to the rotation axis. This perpendicular to the beams, passing through the rotation axis plane, provided an mirror symmetry element ofthe components attached to the beams. The role of the rotation axis in the set was provided by an aluminum tube terminated by internal screws on both sides with two A115-34B car alternators. The alternators performed the current transfer function from the support structure to the moving components. The axle weight with all the attached components was distributed over the two alternators': the upper one (supported by a sleeve between the alternator and a screw bolted to the rotor axle) and the lower one (the weight transferred directly by the rotor to the alternator). The two alternators usage were determined by construction considerations and the need of four 400-watt radiant heaters power supply. The power supplying method for the radiant heaters through both alternators was the same. The power grid current was conducted to the brushes attached in the brush holders of the stationary body. The brushes contacted the rotating rotor sliding rings. Wires along the rotating axis conducted electricity from the alternator rotor through the support beams to the lamps.


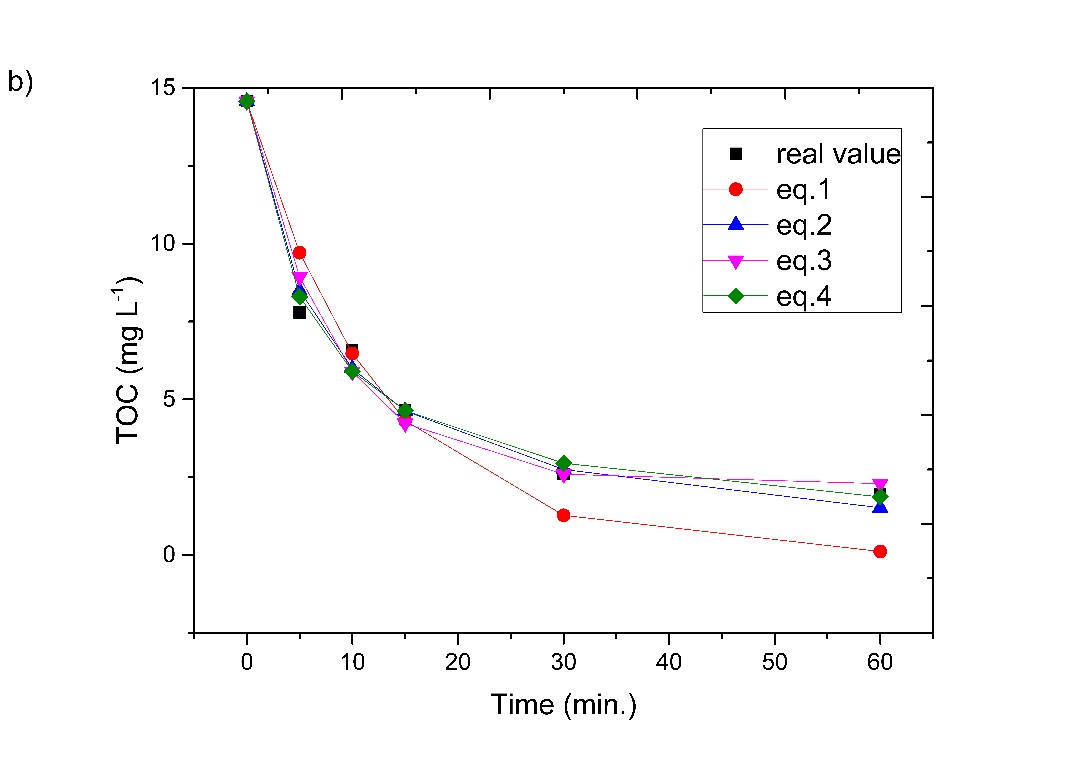


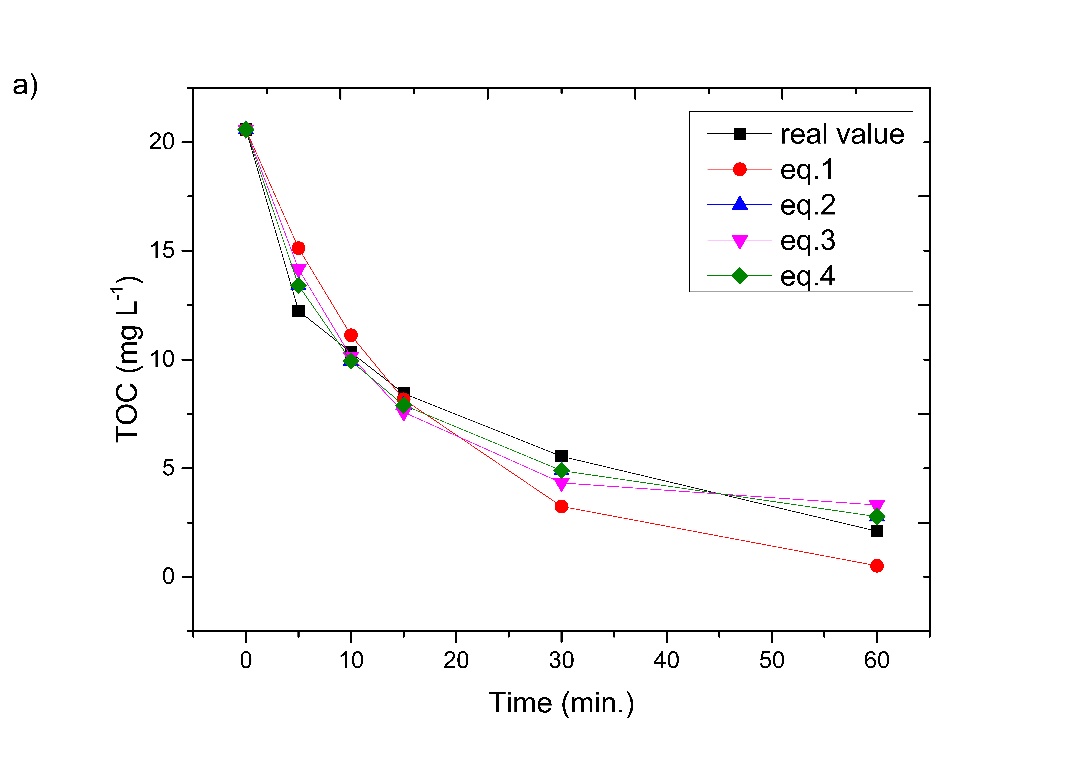


**Figure S3**. Process kinetics with optimal parameters of 400 mg L^-1^ H_2_O_2_, pH3 and UV irradation: a) with 500 mg WI and 50 mg L^-1^ AM E123, b) with 1000 mg WI and 50 mg L^-1^ AM AC.

TOC = TOC_0_ * e^-kt^ [eq.1]

TOC = (kt + 1/TOC_0_)^-1^ [eq.2]

TOC = (TOC_0_ - b) * e^-kt^ + b [eq.3]

TOC = (kt + (TOC_0_ - b)^-1^)^-1^ + b [eq.4]


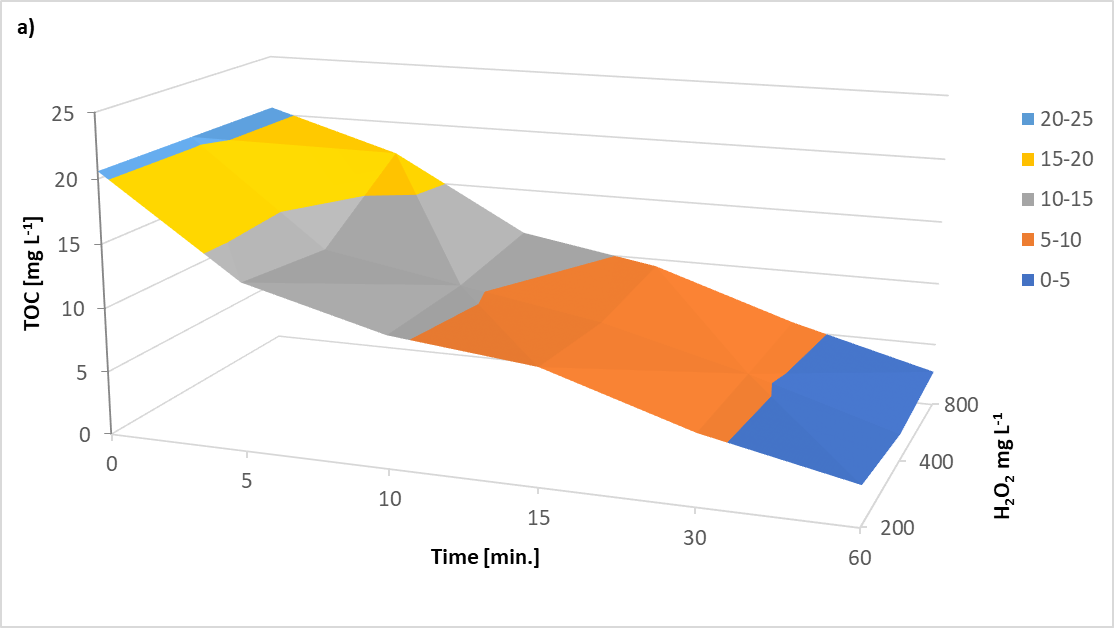


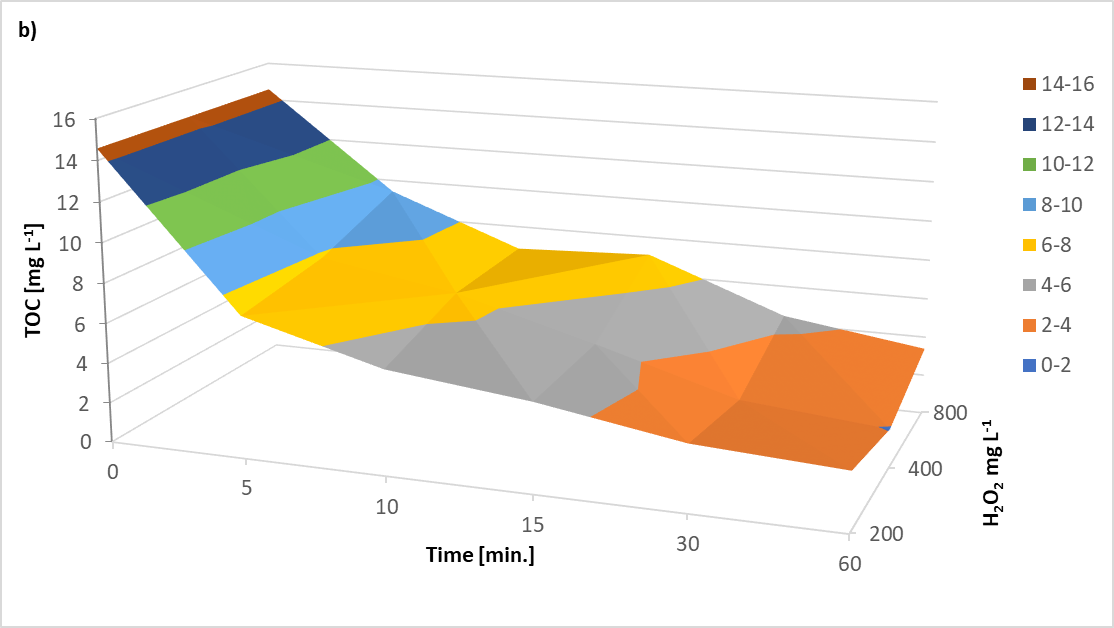


**Figure S4**. ANOVA analises with different H_2_O_2_ doses, pH3 and UV irradation: a) with 500 mg WI and 50 mg L^-1^ AM E123, b) with 1000 mg WI and 50 mg L^-1^ AM AC


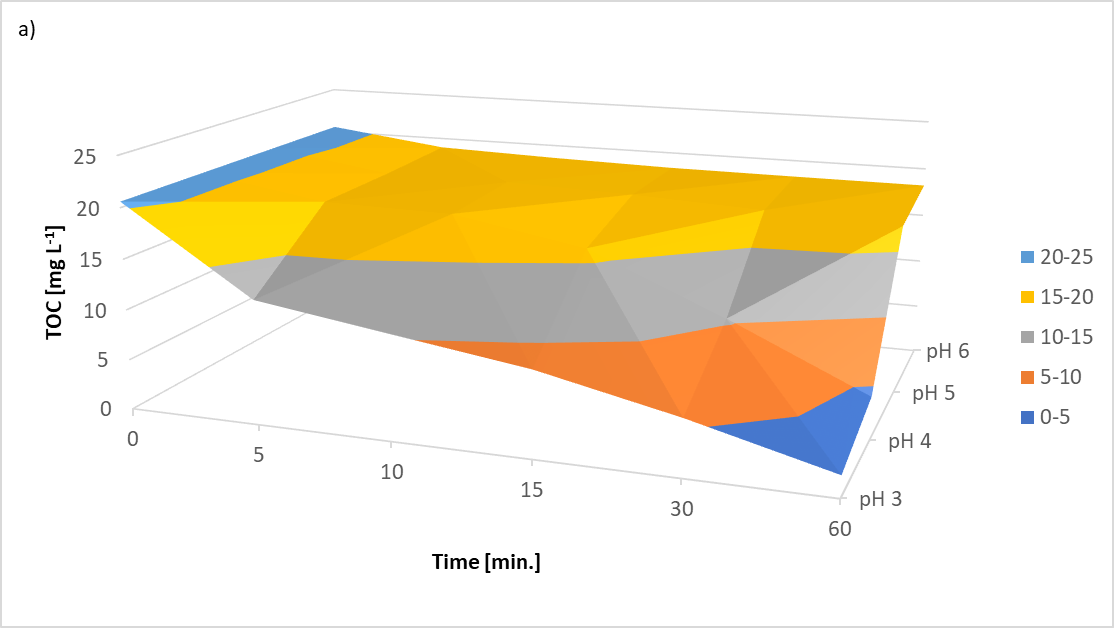


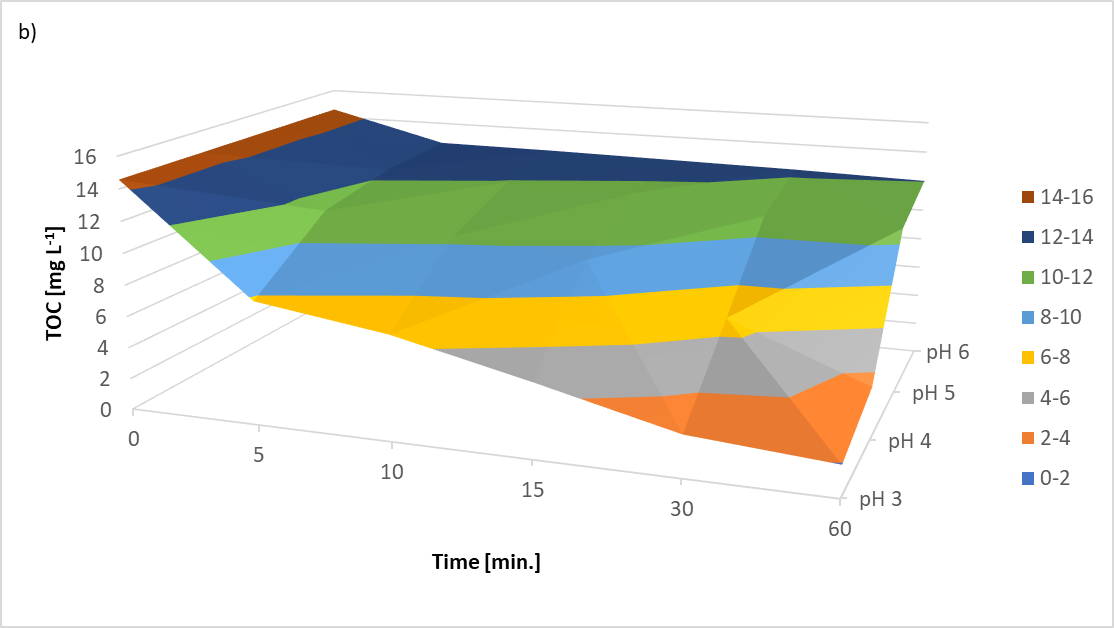


**Figure S5**. ANOVA analises with different pH, 400 mg L^-1^ H_2_O_2_ and UV irradation: a) with 500 mg WI and 50 mgL-1 AM E123, b) with 1000 mg WI and 50 mg L^-1^ AM AC

.


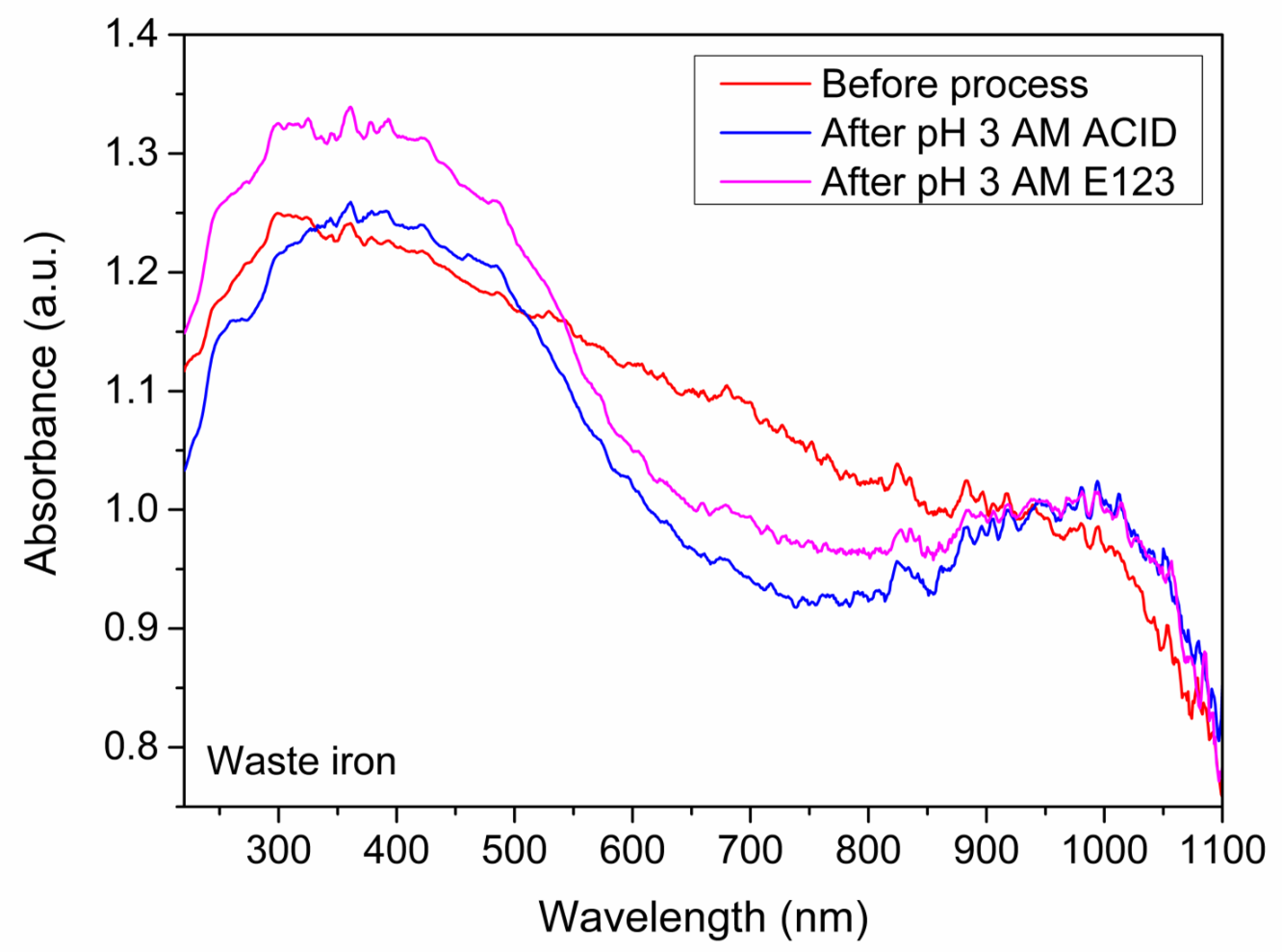


**Figure S6**. The absorbance of the catalyst after 60 min of photo-Fenton process under UV light irradiation.
